# Twenty‐Four‐Hour Urinary Sodium and Potassium Excretion in China: A Systematic Review and Meta‐Analysis

**DOI:** 10.1161/JAHA.119.012923

**Published:** 2019-07-11

**Authors:** Monique Tan, Feng J. He, Changqiong Wang, Graham A. MacGregor

**Affiliations:** ^1^ Wolfson Institute of Preventive Medicine Barts and The London School of Medicine & Dentistry Queen Mary University of London London United Kingdom

**Keywords:** 24‐hour urinary excretion, China, meta‐analysis, potassium, sodium, Diet and Nutrition, Primary Prevention, Risk Factors

## Abstract

**Background:**

In China, high sodium and low potassium intakes result in elevated blood pressure, a major cause of cardiovascular disease, yet the intake estimates lack accuracy and nutritional strategies remain limited.

**Methods and Results:**

We aimed to determine sodium and potassium intake by systematically searching for and quantitatively summarizing all published 24‐hour urinary sodium and potassium data (ie, the most accurate method). MEDLINE, EMBASE, Scopus, China National Knowledge Infrastructure, and Wanfang were searched up to February 2019. All studies reporting 24‐hour urinary sodium or potassium in China were included; hospitalized patients were excluded. Data were pooled using random‐effects meta‐analysis and heterogeneity was explored with meta‐regression. Sodium data were reported in 70 studies (n=26 767), 59 of which also reported potassium (n=24 738). Mean sodium and potassium excretions were 86.99 mmol/24 h (95% CI, 69.88–104.10) and 14.65 mmol/24 h (95% CI, 11.10–18.20) in children aged 3 to 6 years, 151.09 mmol/24 h (95% CI, 131.55–170.63) and 25.23 mmol/24 h (95% CI, 22.37–28.10) in children aged 6 to 16 years, and 189.07 mmol/24 h (95% CI, 182.14–195.99) and 36.35 mmol/24 h (95% CI, 35.11–37.59) in adults aged >16 years. Compared with southern China, sodium intake was higher in northern China (*P*<0.0001) but is declining (*P*=0.0066).

**Conclusions:**

Average sodium intake in all age groups across China is approximately double the recommended maximum limits, and potassium intake is less than half that recommended. Despite a decline, sodium intake in northern China is still among the highest in the world, and the North–South divide persists. Urgent action is needed to simultaneously reduce sodium and increase potassium intake across China.

**Clinical Perspective**

**What Is New?**

- Our study is the first to have systematically assessed and pooled all published 24‐hour urinary sodium and potassium data (ie, the most accurate method to estimate sodium and potassium intake) in China.
- We found that (1) sodium intake in Chinese children, adolescents, and adults has been among the highest in the world over the past 4 decades; (2) the North–South divide in sodium intake still exists, despite there being a decline in northern China; and (3) potassium intake in all age groups has been consistently low throughout the country.

**What Are the Clinical Implications?**

- A coherent, workable, and nationwide strategy is urgently needed in China to simultaneously speed up the pace of sodium reduction and increase potassium intake.
- One way to achieve this dual objective is to replace regular salt with low‐sodium, high‐potassium salt substitutes, which have been shown to lower blood pressure and reduce cardiovascular mortality in randomized trials.

A high‐sodium, low‐potassium diet leads to elevated blood pressure and ultimately cardiovascular disease,^1^, ^2^, ^3^ which is the major cause of death and disability in China and the rest of the world.^4^, ^5^ The World Health Organization recommends that all adults reduce their sodium intake to <87 mmol (<5 g of salt) per day and increase their potassium intake to ≥90 mmol (≥3.5 g) per day, and the recommendations for children are adjusted for their energy requirements and age.^6^, ^7^ In China, the average diet contains too much sodium and not enough potassium,^8^ and strategies to address this situation remain limited.^9^ Moreover, current figures for sodium and potassium intakes in China lack accuracy, as they are often estimated with unreliable methods, such as dietary recalls, food records, or spot urines. The most accurate way to assess sodium and potassium intake is 24‐hour urine collection.^10^, ^11^ A great number of studies have reported such data in China, but there has been no systematic review to comprehensively assess them.

Given the large share of the world's cardiovascular disease burden borne by China—particularly in the form of elevated blood pressure and stroke—and the need to track progress on global targets, more robust estimates of sodium and potassium intake are urgently needed. Therefore, our study aimed to determine sodium and potassium intake in China by systematically searching for and quantitatively summarizing all published data on 24‐hour urinary sodium and potassium excretion in children and adults.

## Methods

The authors declare that all supporting data are available within the article and its online supplementary files.

### Search Strategy and Selection Criteria

We performed a systematic review and meta‐analysis of the published literature. Studies were eligible for inclusion if they were conducted in China and reported summary measurements of 24‐hour urinary sodium or potassium excretion. There was no restriction on study year, design, or language. For hospital‐based studies, only healthy participants were included.

MEDLINE (from 1950 to February 1, 2019), EMBASE (from 1950 to February 1, 2019), Scopus (from 1980 to February 1, 2019), the China National Knowledge Infrastructure (from 1979 to February 1, 2019), and WanFang (unclear start date to February 1, 2019) were searched. The following search terms were used for MEDLINE and subsequently adapted for the other electronic databases (Data S1), with explosion whenever possible:

1. exp Sodium Chloride/OR exp Sodium/OR salt.mp OR exp Potassium/.
2. exp China/OR Chinese.mp OR exp Taiwan/.
3. dietary.mp OR intake.mp OR urinary.mp.
4. 1 AND 2 AND 3.

The reference lists of relevant articles and reviews^12^, ^13^, ^14^, ^15^ were manually searched to identify any other eligible studies. The literature search, data extraction, and risk of bias assessment were carried out independently by 2 authors (M.T. and C.W.). Disagreements were resolved with the help of the other authors.

### Data Extraction and Analysis

Using a spreadsheet, we extracted data on participants characteristics, sample size, age, sex, geographic location, region type (urban versus rural), study design, dates and methods of data collection, the 24‐hour urinary excretions of sodium, potassium, creatinine, and 24‐hour urine volume (mean, SD, SEM). When information was missing, study authors were contacted; if left unanswered, the following assumptions were made: study sites were based on the authors’ affiliations (n=3),^16^, ^17^, ^18^ and study years were assumed to be 3 years before publication (n=20).^16^, ^17^, ^18^, ^19^, ^20^, ^21^, ^22^, ^23^, ^24^, ^25^, ^26^, ^27^, ^28^, ^29^, ^30^, ^31^, ^32^, ^33^, ^34^, ^35^ All measures were converted into millimoles of sodium and potassium using standard conversion values (1 mmol sodium=1 mEq sodium=23 mg sodium; 1 mmol potassium=1 mEq potassium=39.1 mg potassium). If SEM was not reported, it was calculated from the SD and the number of participants. If the period of data collection covered more than a year, the midpoint was used. If several publications reported the same study, only the publication that provided the most data was selected. In interventional studies, if both baseline and end‐of‐trial measurements were reported, only the former were used (n=4).^20^, ^36^, ^37^, ^38^

The risk of bias within each study was assessed using an adapted version of a critical appraisal checklist developed for systematic reviews of prevalence (Data S2).^39^ The checklist consisted of 9 questions related to the quality of sampling, reporting, measurement, analysis, and response rate. We did not formally assess for publication bias and selective outcome reporting because such a bias was highly unlikely, as the 24‐hour urinary excretions were reported either in observational studies or as secondary outcomes.

Data were pooled using random‐effects meta‐analysis. Subgroup analyses were performed to determine sodium and potassium excretion by age group, sex, geographic location, study year, and rigor of 24‐hour urine collection (24‐hour collection was considered rigorous if its completeness was assessed). Evidence for differences in excretion according to these covariates was sought using meta‐regression analyses. Because of small sample sizes, only univariate meta‐regression analyses were performed when northern and southern China were analyzed separately. We used a North–South demarcation of China that was determined by a spatial analysis using geographic information system, based on a model of climate‐, geography‐, and human‐related indicators.^40^ Although prespecified, no subgrouping by region type was made, as only 2 studies reported urban and rural data separately.^41^, ^42^ Neither subgroup nor meta‐regression analyses were performed on studies conducted in children because of the small number of studies available. A 2‐sided *P* value of <0.05 was considered significant. All analyses were performed using R (version 3.4.3) with the packages “meta” (version 4.9‐3) and “metafor” (version 1.9‐9).

## Results

**Figure 1:**
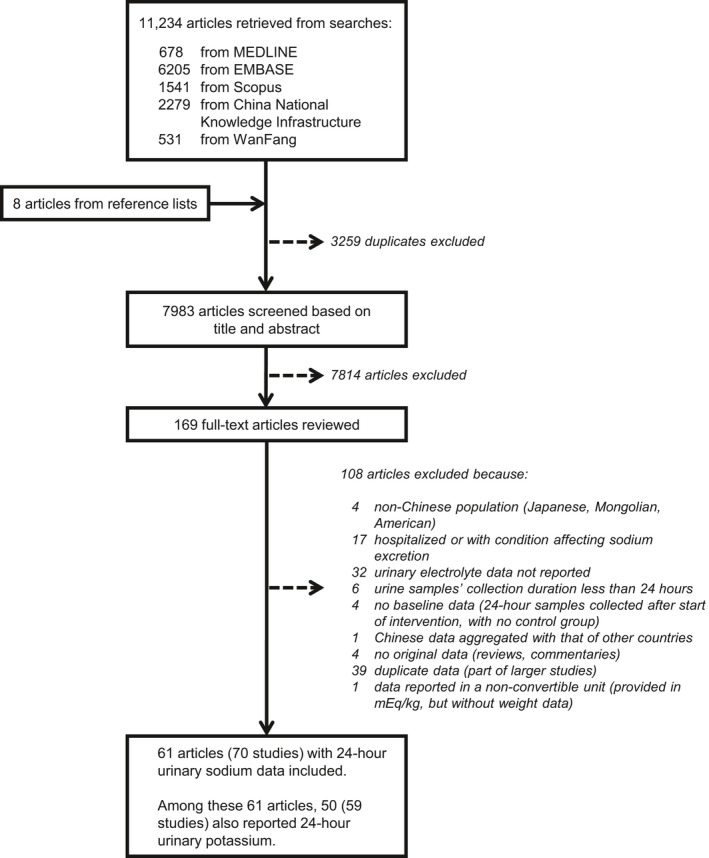
Study selection.

Our search found 11 234 records. After removing the duplicates and searching the reference lists of relevant papers,^12^, ^13^, ^14^, ^15^ 7983 abstracts were screened, and 169 publications were selected for full‐text review, of which 108 were excluded for reasons summarized in Figure 1. A total of 61 papers met the inclusion criteria and were included in our meta‐analysis.^16^, ^17^, ^18^, ^19^, ^20^, ^21^, ^22^, ^23^, ^24^, ^25^, ^26^, ^27^, ^28^, ^29^, ^30^, ^31^, ^32^, ^33^, ^34^, ^35^, ^36^, ^37^, ^38^, ^41^, ^42^, ^43^, ^44^, ^45^, ^46^, ^47^, ^48^, ^49^, ^50^, ^51^, ^52^, ^53^, ^54^, ^55^, ^56^, ^57^, ^58^, ^59^, ^60^, ^61^, ^62^, ^63^, ^64^, ^65^, ^66^, ^67^, ^68^, ^69^, ^70^, ^71^, ^72^, ^73^, ^74^, ^75^, ^76^, ^77^, ^78^, ^79^ Two multisite studies reported separate estimates for each location,^45^, ^49^ and we treated each site as an individual study so as not to lose geographic information. As such, we included a total of 70 studies reporting 24‐hour urinary sodium data (drawn from 890 children: 56% boys, mean age 9 years; and 25 877 adults: 50% men, mean age 46.3 years). Among the 70 studies, 59 also reported 24‐hour urinary potassium data (drawn from 831 children, 56% boys, mean age 8.1 years; and 23 907 adults; 51% men, mean age 46.5 years). There was no study that reported 24‐hour urinary potassium data without 24‐hour urinary sodium data. The data spanned 1981 to 2016 and covered 27 of the 33 administrative regions (provinces, autonomous regions, municipalities, and special administrative regions) of China. Only one 24‐hour urine was collected per participant in 76% (n=53) of the studies reporting sodium data and 75% (n=44) of the studies with potassium data. Data collection was considered rigorous in 51% (n=36) of the studies reporting sodium data and 58% (n=34) of the studies with potassium data. The characteristics of the included studies and participants are provided in Table S1. The risk of bias of each study varied substantially across criteria (Figure S1).

**Figure 2:**
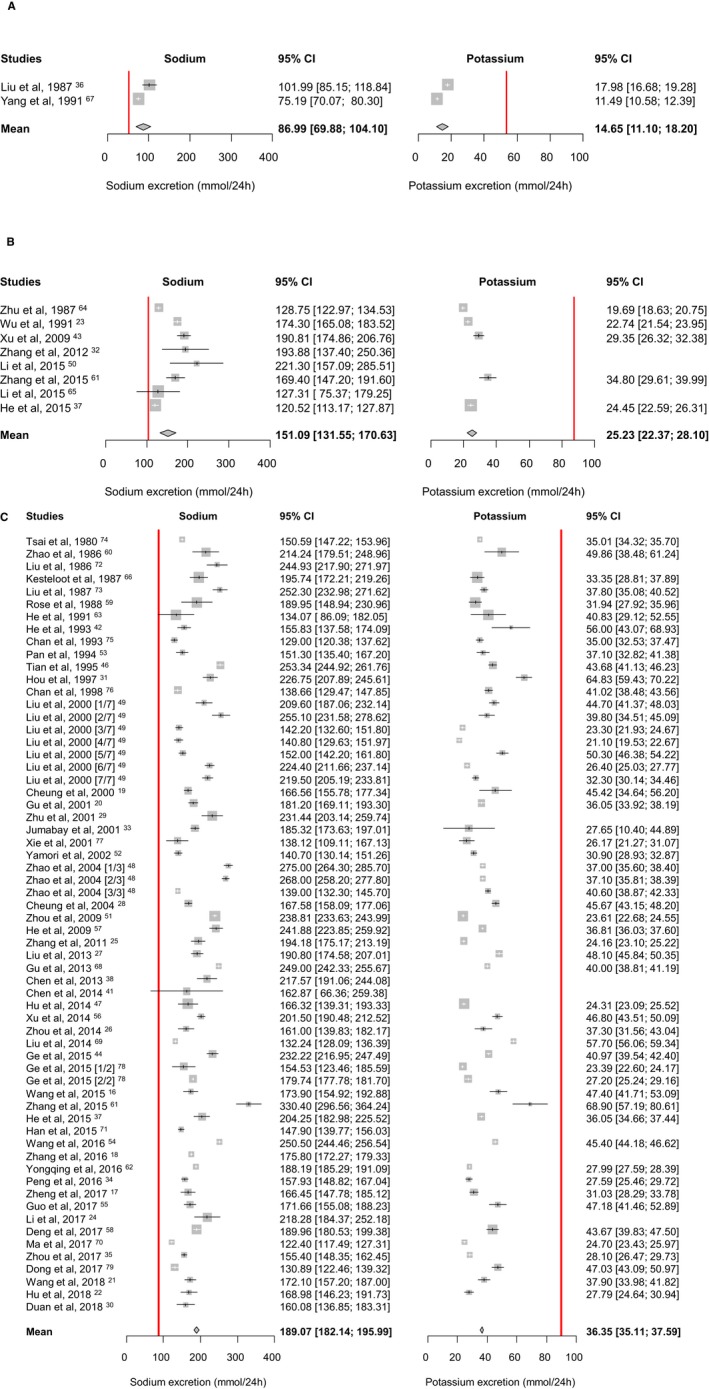
Mean urinary sodium and potassium excretion (mmol/24 h) by age groups. **A**, Aged 3–6 years; **B**, Aged 6–16 years; **C**, Aged ≥16 years. The red lines denote the recommended intakes for children (Chinese Proposed Intakes for Preventing Non‐communicable Chronic Disease for 4–6 and 11–13 year‐olds, respectively^81^, ^82^) and adults (World Health Organization recommendations^6^, ^7^).

In children aged 3 to 6 years, mean sodium excretion was 86.99 mmol/24 h (95% CI, 69.88–104.10), and mean potassium excretion was 14.65 mmol/24 h (95% CI, 11.10–18.20). In children aged 6 to 16 years, mean sodium excretion was 151.09 mmol/24 h (95% CI, 131.55–170.63) and mean potassium excretion was 25.23 mmol/24 h (95% CI, 22.37–28.10). In adults aged 16 years and above, mean sodium excretion was 189.07 mmol/24 h (95% CI, 182.14–195.99), and mean potassium excretion was 36.35 mmol/24 h (95% CI, 35.11–37.59) (Figure 2). Mean creatinine excretion in adults, as reported in 25 studies, was 8.69 mmol/24 h (95% CI, 8.16–9.22). Mean urine volume in adults, as reported in 16 studies, was 1447 mL (95% CI, 1408–1486).

All results reported thereafter pertain to adults only. In men, mean sodium excretion was 194.76 mmol/24 h (95% CI, 179.27–210.25) and mean potassium excretion was 38.26 mmol/24 h (95% CI, 35.65–40.86). In women, mean sodium excretion was 181.54 mmol/24 h (95% CI, 167.10–195.99) and mean potassium excretion was 36.76 mmol/24 h (95% CI, 33.37–40.15). Among studies in which the 24‐hour urine was assessed for completeness, the mean excretions were 188.04 mmol/24 h (95% CI, 175.56–200.52) for sodium and 37.45 mmol/24 h (95% CI, 34.55–40.34) for potassium. Among studies where completeness assessment was not performed or not reported, the mean excretions were 188.43 mmol/24 h (95% CI, 172.96–203.90) for sodium and 37.13 mmol/24 h (95% CI, 33.53–40.73) for potassium.

**Figure 3:**
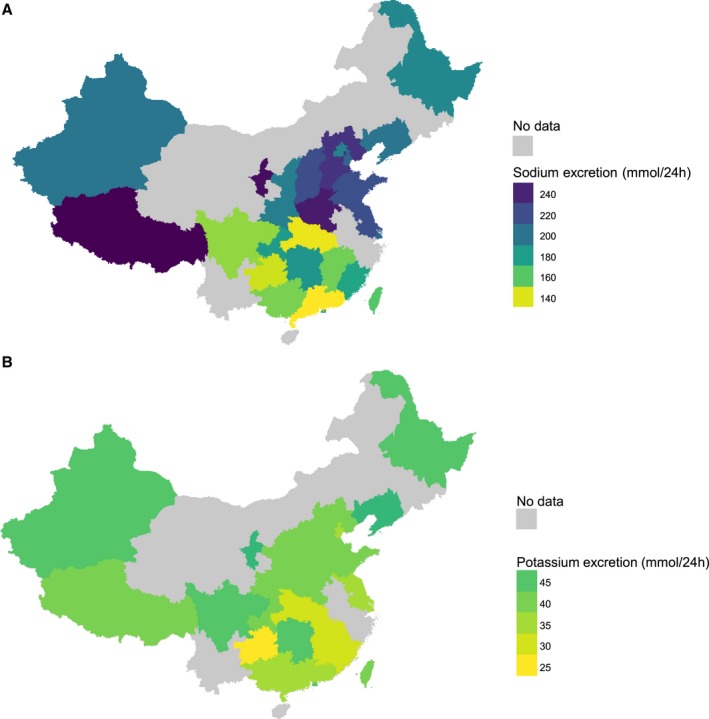
Mean urinary sodium and potassium excretion (mmol/24 h) in adults, per administrative region. **A**, Sodium; **B**, Potassium.

There was a geographic pattern in the 24‐hour urinary excretion of sodium (but not of potassium), with the highest sodium excretions found in northern China: 255.10 mmol/24 h (95% CI, 231.58–278.62) in the Tibet Autonomous Region, 250.50 mmol/24 h (95% CI, 236.99–264.01) in the Ningxia Hui Autonomous Region, and 243.79 mmol/24 h (95% CI, 230.96–256.62) in Henan Province; whereas the lowest sodium excretions were found in southern China: 135.75 mmol/24 h (95% CI, 125.11–146.38) in Guangdong Province, 138.12 mmol/24 h (95% CI, 109.11–167.13) in Hubei Province, and 142.20 mmol/24 h (95% CI, 132.60–151.80) in Guizhou Province (Figure 3). In meta‐regression analyses, there was a significant association between sodium excretion and geographic location, which remained significant (*P*<0.0001) after adjusting for age, sex, study year, and rigor of 24‐hour urine collection (Table).

**Table 1:** Potential Effect Modifiers of Sodium and Potassium Excretion (mmol/24 h) in Adults

	Sodium				Potassium			
	Univariate		Multivariate		Univariate		Multivariate	
	Slope (95% CI)	*P* Value	Slope (95% CI)	*P* Value	Slope (95% CI)	*P* Value	Slope (95% CI)	*P* Value
Age, y	0.25 (−0.59 to 1.09)	0.5573	0.08 (−0.77 to 0.94)	0.8491	0.02 (−0.16 to 0.2)	0.8311	−0.07 (−0.31 to 0.16)	0.5363
Sex (% men)	0.53 (0.04–1.01)	0.0337	0.27 (−0.23 to 0.76)	0.2853	0.01 (−0.11 to 0.13)	0.9054	0.00 (−0.15 to 0.15)	0.9900
Geographic location (each administrative region coded from south to north)	3.25 (2.24–4.27)	<0.0001	3.15 (1.98–4.32)	<0.0001	0.15 (−0.1 to 0.41)	0.2339	0.15 (−0.16 to 0.46)	0.3348
Rigor of 24‐hour urine collection (not rigorous or not reported as reference)	−12.47 (−30.22 to 5.29)	0.1665	−0.24 (−1.04 to 0.56)	0.5493	1.18 (−2.68 to 5.04)	0.5444	1.84 (−2.53 to 6.21)	0.4041
Year of data collection (whole of China)	0.18 (−0.64 to 0.99)	0.6723	−7.85 (−24.63 to 8.92)	0.3547	0.11 (−0.07 to 0.29)	0.2191	0.10 (−0.10 to 0.30)	0.3252
Year of data collection (northern China only)	−1.30 (−2.23 to −0.38)	0.0066	···	···	−0.01 (−0.24 to 0.22)	0.9284	···	···
Year of data collection (southern China only)	1.08 (0.04–2.13)	0.0422	···	···	0.20 (−0.10 to 0.51)	0.1866	···	···

**Figure 4:**
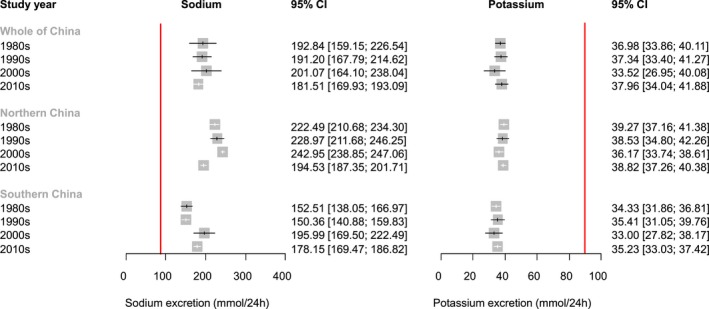
Mean urinary sodium and potassium excretion (mmol/24 h) in adults, per decade of data collection. The red lines denote the World Health Organization–recommended intakes for adults.^6^, ^7^

**Figure 5:**
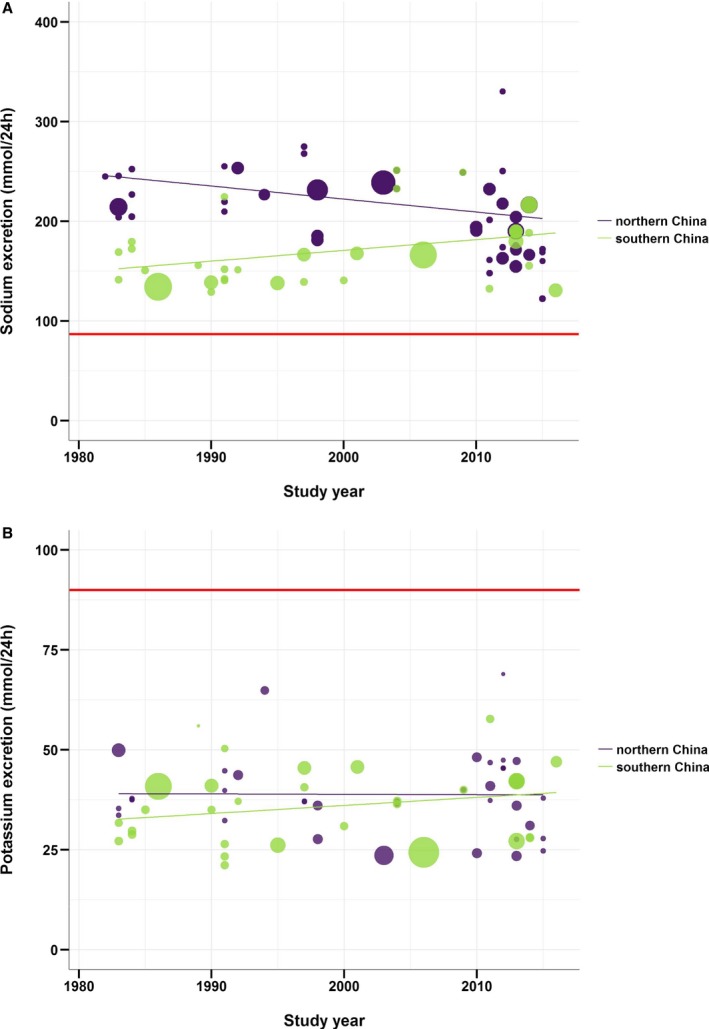
Time trends in adults’ mean 24‐hour urinary sodium and potassium excretion. **A**, Sodium; **B**, Potassium. The red lines denote the World Health Organization–recommended intakes for adults.^6^, ^7^

To examine time trends in sodium and potassium excretion, we pooled the estimates per decade of data collection (Figure 4). While no time trend was apparent when considering China as a whole, subgrouping by region showed that mean sodium excretion decreased in northern China (most markedly between the 2000s and the 2010s) and increased in southern China (most markedly between the 1990s to the 2000s). Both time trends in sodium excretion were confirmed in meta‐regression analyses in which study year was treated as a continuous variable (*P*=0.0066 and 0.0422, respectively). In contrast, potassium excretion has remained stable in both northern and southern China over the past 4 decades (Table, Figure 5).

Sensitivity analyses were carried out by excluding hospital‐based studies, hypertensive participants, and participants belonging to ethnic minority groups (Uygur, Kazakh, Tibetan, Yi, She). We also reran all analyses placing the Tibet Autonomous Region in southern instead of northern China, as the spatial calculation we used to demarcate the country left ambiguity for this area.^40^ All findings remained unchanged, except for the trend of increase in sodium excretion in southern China, which was no longer significant (Tables S2 through S5).

## Discussion

To our knowledge, this is by far the most comprehensive systematic review and meta‐analysis that included all studies using the most accurate method of sodium and potassium intake assessment (ie, 24‐hour urinary excretion) and covering almost all geographic locations across China. Data from 26 767 participants were used to determine sodium intake, which has been consistently high over the past 4 decades and with a North–South divide that persists despite there being a decline in northern China. Data on potassium were also reported for 24 738 participants, revealing consistently low intake levels across the country.

In their respective age group, sodium intake levels in China exceeded all recommendations (with adults consuming double their recommended maximum intake)^6^, ^81^ and were among the highest in the world.^12^, ^15^ In contrast, potassium intake levels were less than half the recommended minimum intake for each age group.^7^, ^82^

Because of the exclusive use of 24‐hour urinary data, our estimates are more robust than previous ones. It is well known that dietary methods are unreliable for the assessment of sodium and potassium intake.^10^ Most of the sodium in the Chinese diet comes from the salt added during home cooking or at the table,^8^ and this discretionary salt use is highly variable and difficult to quantify by dietary methods.^10^ Furthermore, processed and out‐of‐home foods are increasingly consumed in all sociodemographic groups,^8^ but their sodium content tends to be inaccurately reported in food composition tables, and they are also impractical to record^10^—to the extent that out‐of‐home meals were altogether excluded from some previous reports.^83^ The China Health and Nutrition Survey found that the main food sources of potassium in China were wheat products, rice, and potatoes.^8^ The potassium content of such foods vary greatly depending on their preparation, cooking, and processing,^84^, ^85^ which dietary surveys and food composition tables often fail to capture. The use of spot urines has also been repeatedly shown to be unreliable in estimating sodium and potassium intake.^80^, ^86^, ^87^, ^88^ This is mostly attributable to the variation in the excretion of sodium and potassium throughout the day as well as the use of formulas to extrapolate their concentrations to 24 hours, which introduces a source of systematic error.^80^, ^89^

The geographic patterns shown in our study are in agreement with those found in the China Health and Nutrition Survey.^8^ While no major regional difference was apparent for potassium intake, there was a North–South gap in sodium intake. This gap has been documented since the 1980s^8^, ^90^, ^91^, ^92^, ^93^ but may be closing.^14^ Our results suggested a decline in sodium intake in northern China, most markedly since the 2000s. This is likely to be the result of both governmental efforts in salt awareness education and the lessened reliance on pickles attributable to a greater year‐round availability of vegetables,^8^, ^14^, ^94^ although this did not translate into an increase in potassium intake. This trend of decreased sodium intake was not seen in southern China. This could be attributable to the growing consumption of processed foods and out‐of‐home meals, which could ultimately offset any decline in sodium intake achieved so far.^83^ These trends partially contradict those of dietary‐based studies, all of which found large declines in sodium intake across the entire country of China, at both the national^8^, ^83^, ^95^ and the regional^8^ levels. This discrepancy reflects the major limitations of dietary assessment methods, which are likely to have overestimated sodium intake in the past and underestimated it more recently. When food supplies were limited and refrigerator ownership was low, salt was the major food preservative. Older studies conducted during periods of heavy salting recorded all the salt used, even though most of it would eventually be discarded. Recent underestimates are linked to the increasing contribution of processed and out‐of‐home foods to sodium intake, as previously discussed. Further highlighting their unreliability, when different dietary methods were simultaneously and repeatedly used in the same provinces, opposite time trends in sodium intake were obtained in some areas.^83^

Of note, the China Health and Nutrition Survey recorded the highest sodium intakes in the provinces of Shandong, Jiangsu, and Henan, which they considered “central” China.^8^ In our study, these provinces were considered to belong to “northern” China. Such inconsistency is common, as the division of China into regions often seems arbitrary. We opted for a more robust North–South demarcation.^40^ To minimize the impact of our choice, we treated geographic location as a continuous variable in our meta‐regression analysis by coding each administrative region from the farthest south to the farthest north using their longitude. This analysis confirmed a gradient in sodium intake, increasing from the south to the north.

The main strength of our study resides in the comprehensiveness of its search strategy: We used broad search terms that we exploded whenever possible; we searched both Western and Chinese databases (which have been shown to have little overlap^96^, ^97^); and there was no restriction on study year, design, or language. We identified up to 10 times more articles reporting 24‐hour urinary sodium data in China than previous reviews (1 review included 57 articles, but 52 of them reported estimates by dietary methods^14^),^12^, ^13^, ^14^, ^15^ resulting in a broader time and country coverage as well as a much larger number of participants. To our knowledge, this is the first time 24‐hour urinary potassium data in China have been reviewed. This is also the first meta‐analysis of children's 24‐hour urinary sodium and potassium excretion in China.

The lack of assessment or report on the completeness of the 24‐hour urine was a limitation. No single standard exists for assessing the completeness of a 24‐hour urine collection, and undercollection is common.^80^ Our estimates were not adjusted for nonurinary (eg, feces, sweat) losses. Therefore, our figures are underestimates of the true sodium and potassium intakes in China. Other domains with high risks of bias (sample size calculation, sampling frame, calculation of sodium and potassium excretion) reflected reporting rather than study quality and thus did not affect our findings. Finally, the data available did not allow for province‐level comparisons over time; the time trends in our report should be interpreted at the regional level.

Although sodium intake is suggested to have decreased in northern China, the most recent data show that the intake level is still more than double the maximum intake recommended by the World Health Organization; while in southern China, there is a trend of increase. Urgent action is required to accelerate sodium reduction in all regions of China. The Chinese government has made sodium reduction a key component of “Healthy Lifestyle for All,” an initiative to prevent non‐communicable diseases. An action group, “Action on Salt China,” has taken up the task of harnessing support and participation from all regions across China to develop tailored and sustainable sodium‐reduction interventions.^98^ The rapid increase in the consumption of processed and out‐of‐home foods must be addressed before the hard‐won declines in sodium intake are offset. Nevertheless, discretionary salt use still constitutes the vast majority of the sodium consumed in China. Behavior change thus remains primordial, and key periods for the formation of dietary habits are childhood and adolescence. Reducing children's sodium intake leads to a decrease in their blood pressure, which could prevent hypertension and cardiovascular disease later in life.^99^, ^100^ Replacing regular salt with low‐sodium, high‐potassium salt substitutes would achieve the dual objective of reducing sodium intake while simultaneously increasing potassium intake. Randomized controlled trials have demonstrated the role of salt substitutes in reducing blood pressure and cardiovascular disease mortality.^101^, ^102^, ^103^ Concerns over the risk of hyperkalemia associated with the use of salt substitutes are likely to be unwarranted in the Chinese general population in view of the very low intakes of potassium. Nevertheless, potassium intakes should ideally be increased through foods. Given the sheer size of the Chinese population, achieving sodium reduction together with increasing potassium intake nationwide will result in an enormous benefit for global health.

## Sources of Funding

This research was commissioned by the National Institute for Health Research (NIHR) (NIHR Global Health Research Unit Action on Salt China at Queen Mary University of London) using Official Development Assistance (ODA) funding (16/136/77). Tan and Wang are funded by the NIHR grant, He and MacGregor are partially funded by the NIHR grant. The views expressed in this publication are those of the author(s) and not necessarily those of the NIHR or the Department of Health and Social Care.

## Disclosures

Prof He is a member of the Consensus Action on Salt & Health group, a nonprofit charitable organization, and its international branch, World Action on Salt & Health, and does not receive any financial support from the Consensus Action on Salt & Health or World Action on Salt & Health. Prof MacGregor is the Chairman of Blood Pressure UK, Chairman of the Consensus Action on Salt & Health, and Chairman of World Action on Salt & Health and does not receive any financial support from any of these organizations. Blood Pressure UK, the Consensus Action on Salt & Health, and World Action on Salt & Health are nonprofit charitable organizations. The remaining authors have no disclosures to report.

## Supporting information


**Data S1.** Search strategies.
**Data S2.** Quality analyses of the studies included in the systematic review and meta‐analysis.
**Table S1.** Characteristics of Included Studies
**Table S2.** Mean Sodium Excretion (mmol/24 h) for Subgroups of Studies—Sensitivity Analyses
**Table S3.** Mean Potassium Excretion (mmol/24 h) for Subgroups of Studies—Sensitivity Analyses
**Table S4.** Potential Effect Modifiers of Adults’ Sodium Excretion (mmol/24 h)—Sensitivity Analyses
**Table S5.** Potential Effect Modifiers of Adults’ Potassium Excretion (mmol/24 h)—Sensitivity Analyses
**Figure S1.** Risk of bias in the included studies.
