# Supplementary material for: Twenty‐Four‐Hour Urinary Sodium and Potassium Excretion in China: A Systematic Review and Meta‐Analysis
Source: J Am Heart Assoc. 2019 Jul 11;8(14):e012923. doi: 10.1161/JAHA.119.012923 (PMC6662145; doi:10.1161/JAHA.119.012923)
Supplement: Supplementary file 1 — Data S1. Search strategies. Data S2. Quality analyses of the studies included in the systematic review and meta‐analysis. Table S1. Characteristics of Included Studies Table S2. Mean Sodium Excretion (mmol/24 h) for Subgroups of Studies—Sensitivity Analyses Table S3. Mean Potassium Excretion (mmol/24 h) for Subgroups of Studies—Sensitivity Analyses Table S4. Potential Effect Modifiers of Adults’ Sodium Excretion (mmol/24 h)—Sensitivity Analyses Table S5. Potential Effect Modifiers of Adults’ Potassium Excretion (mmol/24 h)—Sensitivity Analyses Figure S1. Risk of bias in the included studies. [file JAH3-8-e012923-s001.pdf]

SUPPLEMENTAL MATERIAL

Data S1. Search strategies

Database: **MEDLINE** (via Ovid)

Dates: from 1946 to 1st February 2019

Search terms:
1. exp Sodium Chloride/ OR exp Sodium/ OR salt.mp OR exp Potassium/
2. exp China/ OR Chinese.mp OR exp Taiwan/
3. dietary.mp OR intake.mp OR urinary.mp
4. 1 AND 2 AND 3

Database: **EMBASE**

Dates: from 1974 to 1st February 2019

Search terms:
1. 'sodium chloride'/exp OR 'sodium chloride' OR 'sodium'/exp OR 'sodium' OR ‘salt’ OR ‘potassium’/exp OR ‘potassium’
2. 'china'/exp OR 'china' OR 'chinese'/exp OR 'chinese' OR 'taiwan'/exp OR 'taiwan'
3. 'dietary' OR 'intake' OR 'urinary'
4. 1 AND 2 AND 3

Database: **Scopus**

Dates: from 1980 to 1st February 2019

Search terms:
1. 'sodium chloride' OR 'sodium' OR ‘salt’ OR ‘potassium
2. 'china' OR 'chinese' OR 'taiwan'

3. 'dietary' OR 'intake' OR 'urinary'

4. 1 AND 2 AND 3

Database: **China National Knowledge Infrastructure (CNKI)**

Dates: from 1979 to 1st February 2019

Search terms: 1. SU = ('盐' + '钠' + '食用盐' + '食用钠' + '钾')

2. SU = ('中国' + '中国人' + '我国' + '台湾')

3. SU = ('消耗' + '摄入' + '食用' + '尿')

4. 1 * 2 * 3

Database: **WanFang**

Dates: unclear start date, to 1st February 2019

Search terms: 1. 主题: ('盐' + '钠' + '食用盐' + '食用钠' + '钾')

2. 主题: ('中国' + '中国人' + '我国' + '台湾')

3. 主题: ('消耗' + '摄入' + '食用' + '尿')

4. 1 * 2 * 3

Data S2. Quality analyses of the studies included in the systematic review and meta-analysis

- Appropriate sample frame? Whether the sample frame was appropriate to address the target population (eg, if the target population consisted of the Chinese adult population, choosing the outpatient department of a single hospital was not considered an appropriate sampling frame and was therefore marked as 'high risk').
- Appropriate sampling method**?** Whether the sample was representative of the population (eg, convenience samples were not considered appropriate and was therefore marked as 'high risk').
- Adequate sample size (sample size calculation)? Whether a sample size calculation to detect a difference in sodium or potassium intake was made (eg, a sample size calculation to detect a difference in systolic blood pressure was not considered to be appropriate and was therefore marked as 'high risk').
- Detailed description of subjects and setting? Whether the study sample was described in sufficient details so that other researchers can determine if it is comparable to the population of interest to them (eg, not providing study sites was not to be considered appropriate and was therefore marked as 'high risk').
- Sufficient coverage of the data analysis? Coverage bias: whether all subgroups of the identified sample responded at the same rate (if separate response rates were not provided for the different subgroups of the sample, this was marked as 'unclear risk').
- Valid data collection methods (completeness of samples assessed)? Measurement bias: whether the completeness of the urine samples was assessed or not (if not reported in the article, this was marked as 'unclear risk').
- Standardised data collection (staff trained, instructions given)? Whether the study staff were trained in 24h urine samples collection and whether the participants were given

clear instructions for collection (both criteria had to be met for this domain to be marked as ‘low risk’)

- Appropriate calculation of sodium or potassium excretion? Whether enough details were provided on how the sodium or potassium excretion values were obtained from the urine samples.
- Adequate response rate? Whether there was a minimum of 80% response rate (if not, this was marked as ‘high risk’).

Table S1. Characteristics of included studies

Children aged 3–6 years

**Study**	**Design**	**Site**	**Study dates**	**Population**		**n**	**Sodium excretion ±SE, mmol/24h**	**Potassium excretion ±SE, mmol/24h**	**Creatinine excretion ±SE, mmol/24h**	**Urine volume ±SE, ml/24h**	**Assessment of 24h urine completeness**
Liu et al, 1987[1]	Non-randomised controlled trial	Two kindergartens of the Capital Iron and Steel Company, Beijing	Apr-May 1984	Children aged 3-5 years	Experimental group (baseline)	36	108.9±4.39	18.3±0.9	2.02±0.15		NR
					Control group (baseline)	37	91.3±9.27	17.6±0.98	1.24±0.1		
Yang et al, 1991[2]	Pre-post trial	Wuhan	NR	Children aged 4-6 years	Before saline load: family history of essential hypertension	35	71.89±3.89	11.28±0.72			NR
					Before saline load: without family history of essential hypertension	51	77.25±2.92	11.63±0.6			

Children aged 6–16 years

**Study**	**Design**	**Site**	**Study dates**	**Population**		**n**	**Sodium excretion ±SE, mmol/24h**	**Potassium excretion ±SE, mmol/24h**	**Creatinine excretion ±SE, mmol/24h**	**Urine volume ±SE, ml/24h**	**Assessment of 24h urine completeness**
Zhu et al, 1987[3]	Cross-sectional	Two community primary schools in Wuhan, China	1984-85	Second-grade boys aged 7-8 years		148	128.75±2.95	19.69±0.54	3.66±0.06		Ascertained each day; specimens with 24h creatinine <10 mg/kg body weight discarded and an additional one was collected
			NR		Male	94	181.5±6.69	23.84±0.85			Not assessed

Wu et al, 1991[4]	Cross-sectional	Rural district of Hanzhong municipality, Shaanxi Province		Secondary school students aged 12-16 years	Female	87	166.5±6.55	21.54±0.88			
					Total	181	174.3±4.71	22.74±0.62			
Xu et al, 2009[5]	Cross-sectional	Xinjiang Baliken area	Aug-Sep 2005	Kazakhstan Clan children aged 9-10 years		49	190.81±8.14	29.35±1.55	11.34±0.63		NR
Zhang et al, 2012[6]	Pre-post trial	Department of Pediatrics of Peking University First Hospital	NR	Healthy controls, mean age 13 years (SD=3)	Control group	10	193.88±28.81				NR
Li et al, 2015[7]	Pre-post trial	Peking University First Hospital	Jun 2012-May 2014	Healthy controls, mean age 11. 0years (SD=4.0)	Control group	10	221.3±32.76				NR
Zhang et al, 2015 [children][8]	Cross-sectional	Huairou District, Beijing	Apr 2012	Mean age 10.0 years (SD=3.2) in children, 42.3 years (SD=9.4) in adults	Children	16	169.4±11.33	34.8±2.65	6±1	1037±66	
Li et al, 2015[9]	Cross-sectional	Department of Pediatrics, Peking University First Hospital	Jun 2012-Feb 2014	Children with postural tachycardia syndrome, mean age 11.2 years (SD=2.0); healthy controls, mean age 11.1 years (SD=2.4)	24h Una ≥124 mmol/24h	18	154±5.42				NR
					24h Una ≤124 mmol/24=h	21	101±3.06				
He et al, 2015	Randomised controlled trial	Primary schools in urban	May 2013-Dec 2013	School-EduSalt: fifth-graders (mean age	Control group - children (baseline)	138	116.7±5.2	25.4±0.9	4.9±0.2	862±38	New collection if missed one or more urine voids or spilt

[children][10]		Changzhi, northern China		10.1 years, SD=0.5) and adult members of their families (mean age 43.8 years, SD=12.2)	Intervention group - children (baseline)	140	124.2±5.1	23.5±0.9	4.7±0.2	952±38	>10% of the total 24h urine volume

Adults aged >16 years

Study	Design	Site	Study dates	Population		n	Sodium excretion ±SE, mmol/24h	Potassium excretion ±SE, mmol/24h	Creatinine excretion ±SE, mmol/24h	Urine volume ±SE, ml/24h	Assessment of 24h urine completeness
Tsai et al, 1980[11]	Cross-sectional	Department of Internal Medicine of the National Taiwan University Hospital	Aug 1983-Aug 1987	Healthy controls, aged 39.06 years (SD=2.01)	Healthy controls	36	150.59±1.72	35.01±0.35			Checked by daily urine creatinine excretion
Zhao et al, 1986[12]	Cross-sectional	Xinjiang Autonomous Region	NR	Men aged 40-59 years from communities with little migration and eating traditional foods	Kazak	92	248±9.59	39.9±1.86			NR
					Han	82	188±9.06	57.7±2.93			
					Uygur	83	207±9.55	52.5±2.9			
Liu et al, 1986[13]	Cross-sectional	Fuwai Hospital	1981-83	Healthy male doctors and technicians, aged 30-50 years	1st day	49	231.2±12.84				Each participant was asked to return the specimens with assurance of correct collection. If a mistake was made in the collection, it had to be done over again.
					2nd day	49	249.5±13.3				
					3rd day	49	262.5±13.81				
					4th day	49	236.5±13.94				
					5th day	49	253.5±15.2				
					6th day	49	236.4±13.67				
	Cross-sectional	Northern China:	Nov 1984-	Northern China: mean age 40.4	Men - North	498	226.9±3.98	37.5±0.71	11.11±0.18		Not assessed
					Men - South	504	179.4±3.25	28.8±0.58	11.28±0.14		

Kestelo ot et al, 1987[14]		region of Beijing; South China: region of Fuchow	Jan 1985	years (SD=14.4) in men, 40.2 years (SD=14.4) in women; South China: 40.4 years (SD=14.4) in men, 40.5 years (SD=14.3) in women.	Women - North	505	204.6±3.65	37.5±0.76	7.77±0.13		
					Women - South	501	172.4±3.15	29.7±0.52	7.62±0.09		
Liu et al, 1987[15]	Cross-sectional	Fu-Wai Hospital, Beijing	1984	Healthy normotensive male employees, aged 27-50 years (mean 35)	50	252.3±9.86	37.8±1.39	6±0.16		Not assessed	
Rose et al, 1988[16]	Cross-sectional	Beijing, Nanning, Tianjin, Taiwan	1982-85	INTERSALT: aged 20-59 years	Beijing	200	204.1±4.7	35.3±0.74	9.5±0.11	1370±36	Assessed by a standardised interview
					Nanning	200	169.2±4.32	27.2±0.59	9.4±0.11	1220±36	
					Tianjin	200	245.6±5.89	33.6±0.74	9.6±0.13	1700±42	
					Taiwan	181	141.4±4.47	31.7±1.11	8.7±0.22	1160±36	
He et al, 1991[17]	Cross-sectional	Puge County, Southern China	1986	Yi People Study - four male population groups: high-mountain Yi farmers at ~2,750 m above sea level (mean age 30.9 years, SD=11.5), mountainside Yi farmers at ~1,800 m elevation level (mean age 36.4 years, SD=14.3), Yi people who migrated to the county seat (mean age 39.3 years, SD=12.7), and native Han people of the county seat	High-mountain Yi farmers	119	73.9±4.61	58.6±2.84			Participants questioned about the completeness of the collection by a local physician
					Mountainside Yi farmers	114	117.9±5.19	48.5±2.63			
					County seat Yi migrants	89	159.4±6.64	28.3±1.44			
					County seat Han people	97	186±7.41	29±1.06			

				(mean age 36.4 years, SD=12.1)							
He et al, 1993[18]	Cross-sectional	Liangshan Yi People Autonomous Prefecture (Liangshan), Southwestern China	Apr 1989	Yi Migrant Study: men aged 19-55 years	Rural sample: Yi farmers, day 1	30	119.5±12.56	84±11.05			Subjects asked to repeat collection if reported to be incomplete, or a timing error exceeding 30 minutes was noted
					Rural sample: Yi farmers, day 2	30	136.6±15.06	88.7±13.27			
					Rural sample: Yi farmers, day 3	30	138.3±13	83.2±10.41			
					Urban sample: Yi migrants and Han people, day 1	33	171.6±11.12	29.1±2.12			
					Urban sample: Yi migrants and Han people, day 2	33	172.7±14.41	29.3±2.3			
					Urban sample: Yi migrants and Han people, day 3	33	188.9±9.35	30.7±2.33			
					Total, day 1	63	146.8±8.92	55.2±6.36			
					Total, day 2	63	155.5±10.58	57.6±7.41			
					Total, day 3	63	165.2±8.44	55.2±6.02			
Chan et al, 1993[19]	Cross-sectional	NR	NR	Healthy female university students and visitors of a family clinic, mean age 24.1 years (SD=7.09)		142	129±4.4	35±1.26	7.5±0.17		Not assessed
Pan et al, 1994[20]	Cross-sectional	Taiwan	Mar-Apr 1992	Research staff of the Insitute of Biomedical Sciences, Academia Sinica, in their 20s		40	151.3±8.11	37.1±2.18			NR
Tian et al, 1995[21]	Cross-sectional	Tianjin City	1992	Mean age 43.6 years (SD=13.6) in men, 43.5 years (SD=13.3) in women	Male	328	257.8±4.75	42.4±0.94			
					Female	335	249.2±4.45	45±0.99			
	Pre-post trial	NR	NR	Graduate school students and staff	Salt sensitive subjects	9	221±13.33	66.97±4.04			NR

Hou et al, 1997[22]				members, aged 23-40 years	Non-salt sensitive subjects	14	233±13.9	62.97±3.76			
Chan et al, 1998[23]	Cross-sectional	Hong Kong	Oct 1989-May 1991	Healthy subjects, aged 20-65 years	Men	42	145.2±7.51	40.4±2.33			Undercollection = creatinine output < 5.3 mmol in women and < 7.1 mmol in men; overcollection = creatinine output > 15.9 mmol in women and 17.7 mmol in men
					Women	84	135.3±5	41.3±1.56			
Liu et al, 2000[24]	Cross-sectional	Taiwan, Shanghai, Urumiqi, Lhasa, Guizang, Guangzhou, Shijiazhuang	1985-97	WHO-CARDIAC: aged 48-65 years	Total	1389	189.5±3	32.1±0.6	9.06±0.09		Assessed by urinary creatinine excretion in relation to weight
					Urumigi	200	209.6±11.5	44.7±1.7	9.86±0.44		
					Lhasa	125	255.1±12	39.8±2.7	7.39±0.26		
					Guiyang	206	142.2±4.9	23.3±0.7	7.74±0.18		
					Guangzhou	217	140.8±5.7	21.1±0.8	9.06±0.26		
					Taiwan	200	152±5	50.3±2	10.65±0.35		
					Shanghai	225	224.4±6.5	26.4±0.7	9.77±0.18		
					Shijianzhuang	216	219.5±7.3	32.3±1.1	8.27±0.18		
Cheung et al, 2000[25]	Cross-sectional	Queen Mary Hospital, Hong Kong	NR	Hypertensive outpatients: individuals referred to the hypertension outpatient clinic, mean age 46 years (SD=14); normotensive controls: mean age 41 years (SD=12)	Hypertensive patients - total	70	172±7.65	40±1.91			NR
					Hypertensive patients - male	43	176±10.52	43±2.74			
					Hypertensive patients - female	27	165±10.78	35±2.12			
					Normotensive controls - total	47	161±7.73	51±2.33			
					Normotensive controls - male	21	175±10.47	54±3.49			
					Normotensive controls - female	26	149±10.79	48±3.14			
Gu et al, 2001[26]	Randomised	North of Beijing	NR	Aged 45-64 years	Assigned to potassium	75	175.6±7.44	35.8±1.79	6.25±0.24		NR

	controlled trial				supplementation (baseline)						
					Assigned to placebo (baseline)	75	188±8.37	36.2±1.37	6.83±0.26		
Zhu et al, 2001[27]	Cross-sectional	Department of Cardiology, First Hospital of Xi'an Jiaotong University	NR	Outpatients, mean age 48 years (SD=6) in hypertensives, 47 years (SD=8) in normotensives	High blood pressure - salt sensitive	17	239±17.22				NR
					High blood pressure - salt resistant	15	270±23.75				
					Non-high blood pressure - salt sensitive	8	231±16.97				
					Non-high blood pressure - salt resistant	13	193±18.86				
Jumabay et al, 2001[28]	Cross-sectional	Barkol area in the Xinjiang region	NR	Kazakh and Han people aged 65-70 years	Kazakh subjects	117	181.4±7.17	18.9±0.8			NR
					Han subjects	50	194.1±10.73	36.5±1.6			
Xie et al, 2001[29]	Cross-sectional	Farming village in Hubai Province (North China)	Mar 1995	Mean age 40.0 years (SD=16.5) in men, 36.7 years (SD=15.7) in women	Men	179	152.9±4.67	28.7±1.29	6.6±0.33		Not assessed
					Women	153	123.3±4.79	23.7±1.16	5.1±0.23		
Yamori et al, 2002[30]	Cross-sectional	Daping District of Chongqing	Oct 2000	Extension of WHO-CARDIAC: men aged 43-55 years		118		140.7±5.39	30.9±1		NR
Zhao et al, 2004[31]	Cross-sectional	Pinggu County, Beijing; Yu County, Shanxi Province; Wuming County, Guangxi Zhuang	Sep 1997-Jan 1998	INTERMAP: rural populations, mean age 48.9 years (SD=5.8) in the North, 49.1 years (SD=5.7) in the South	Beijing (North)	272	275±5.46	37±0.72			Specimens rejected if collection time fell outside the range 22-26 h, if the participant responded that collection was incomplete, or he/she had lost 'more than a few
					Shanxi (North)	289	268±5	37.1±0.66			
					North	561	271±3.72	37.1±0.49			
					Guangxi (South)	278	139±3.42	40.6±0.88			

		Autonomous Region									drops' of urine, or if total volume was less than 250 ml. The participant was then asked to repeat the collection.
Cheung et al, 2004[32]	Cross-sectional	Hong Kong	NR	Mean age 40.3 years (SD=12.7) in normotensive subjects, 51.0 years (SD=12.2) in hypertensive subjects	All subjects	190	167.4±4.85	45.7±1.29			Not assessed
					Normotensive	151	166.6±5.57	46.2±1.46			
					Hypertensive	39	170.6±9.8	43.9±2.69			
Zhou et al, 2009[33]	Randomised controlled trial	Rural Hedong District, Tianjin	Sep 2003-May 2004	Rural communities, participants aged 50-80 years	Hypertensives on compound ion salt	62	238±4.89	23.5±0.91			NR
					Hyper-tensivse on normal salt	64	241±5.78	24.6±1.02			
					Normotensives on compoud ion salt	57	237±6.62	22.8±0.95			
					Normotensives on normal salt	65	239±4.54	23.7±0.97			
He et al, 2009[34]	Crossover trial	Rural areas in north China: Hebei, Henan, Shandong, Shaanxi, and Jiangsu provinces	Oct 2003-Jul 2005	GenSalt: mean age 39.3 years (SD=9.6) in men, 38.1 years (SD=9.4) in women	Men	1010	251.1±2.2	37.2±0.3	9.31±0.07		NR
					Women	896	232.7±2.06	36.4±0.33	7.83±0.06		
Zhang et al, 2011[35]	Pre-post trial	Laiwu city, Shandong Province	2010	Rural communities, participants aged 30-60 years	High blood pressure	195	204±4.44	24.8±0.79			NR
					Non-high blood pressure	216	184.6±3.86	23.7±0.65			
Liu et al, 2013[36]	Pre-post trial	Jinxi Second Community Service	NR	Hypertensives Han people, mean	Salt-sensitive hypertensive patients	63	179.47±11.04	47.98±2.47			If incomplete collection, participant asked

		Centre in Chaoyang District, Beijing		age 57.5 years (SD=8.5)	Non-salt-sensitive hypertensive patients	279	196.84±5.48	48.13±1.30			to re-collect the next day
Gu et al, 2013[37]	Crossover trial	18 of the 45 GenSalt study villages in rural areas in northern China	Aug 2008-Nov 2009	Follow-up to the GenSalt study: mean age 44.3 years (SD=8.7)		487	249±3.4	40±0.61			NR
Chen et al, 2013[38]	Randomised controlled trial	Two villages in the suburban area of Beijing	Jun 2012 - Jan 2013	Mean age 54.69 years (SD=12.30) in intervention group, 51.90 years (SD=13.54) in control group	Intervention group (baseline)	99	204.28±10.65				Not assessed
					Control group (baseline)	74	231.34±11.24				
Chen et al, 2014[39]	Cross-sectional	Xicheng and Shunyi districts in Beijing	Jul 2012	Mean age 57.7 years (SD=13.8)	Urban respondents	396	113.7±3.65				NR
					Rural respondents	403	212.18±5.23				
Hu et al, 2014[40]	Cross-sectional	JingNing County	2003-09	Primary study: mean age 46.5 years (SD=15.9) in men, 43.5 years (SD=15.0) in women; age of validation study's participants NR.	Primary study - Gene and polymorphism rs3811544 (NPPC), CC	902	178.4±2.83	24.8±0.34		1240±1	Urinary samples less than 600 mL were excluded
					Primary study - Gene and polymorphism rs3811544 (NPPC), CC±TT	49	205.8±11.6	26.6±1.38		1390±11	
					Validation study - Gene and polymorphism rs3811544 (NPPC), CC	1273	140.2±2.05	23.4±0.35		1110±0	
					Validation study - Gene	82	145.1±8.71	23±1.5		1160±7	

					and polymorphism rs3811544 (NPPC), CC±TT						
Xu et al, 2014[41]	Cross-sectional	Yantai, Shandong Province	Jul 2011	SMASH pilot: mean age 42.3 years (SD=13.5)	Men	98	218.3±8.22	45.9±1.81	11±0.23		Creatinine [mg/day]/body weight [kg] of 14.4 to 33.6 in men and 10.8 to 25.2 in women were classified as indicating an Acceptable 24h urine collection
					Women	93	183.8±7.24	47.7±2.87	7.6±0.17		
					Total	191	201.5±5.62	46.8±1.68	9.4±0.19	1442±42	
Zhou et al, 2014[42]	Crossover trial	NR	NR	Mean age 27.3 years (SD=0.84)	Day 3 (baseline)	23	161±10.8	37.3±2.93	9.73±0.99	1898±38	NR
Liu et al, 2014[43]	Cross-sectional	Hong Kong	2011	Postmenopausal women with prehypertension aged 48-70 years	Sensitivity analyses	569	132.24±2.12	57.7±0.83	8.5±0.11	2082±27	Sensitivity analyses excluded subjects with missed voids and subjects with 30% or higher coefficients of variation in weight-corrected creatinine (24h creatinine excretion in milligrams divided by body weight in kilograms)
Ge et al, 2015[44]	Cross-sectional	Shandong (Gaomi and Fushan sites) and Jiangsu (Xinyi and Ganyu sites)	Jun-Jul 2011	SMASH participants: mean age 39.7 years (SD=13.9) in those without metabolic syndrome, 46.1 years (SD=13.0)	Without metabolic syndrome	1435	224.8±2.12	40.4±0.52			
					With metabolic syndrome	471	240.4±4.05	41.9±0.9			

				in those with metabolic syndrome							
Ge et al, 2015[45]	Cross-sectional	Gaomi and Fushan in Shandong Province, Xinyi and Ganyu in Jiangsu Province	2013	SMASH: mean age 42.1 years (SD=13.4)	Total	2281	166.9±0.54	25.3±0.07			Incomplete urine collection defined as either a 24h urinary volume less than 500 ml or a 24h urinary creatinine volume that was ± 2 SD outside of the sex-specific mean
					Male	1135	172.4±0.86	25.3±0.11			
					Female	1146	161.6±0.65	25.3±0.09			
					Fushan	551	170.4±1.57	23.8±0.18			
					Gaomi	568	138.7±0.99	23±0.12			
					Xinyi	598	178.8±0.75	26.2±0.09			
					Ganyu	564	180.8±0.9	28.2±0.12			
Wang et al, 2015[46]	Crossover trial	Northern China	NR	Rural community, mean age 49.0 years (SD=7.9)	Baseline	48	173.9±9.69	47.4±2.9			NR
Zhang et al, 2015 [adults][8]	Cross-sectional	Huairou District, Beijing	Apr 2012	Mean age 10.0 years (SD=3.2) in children, 42.3 years (SD=9.4) in adults	Adults	10	330.4±17.27	68.9±5.98	13.8±2.9	2079±167	NR
He et al, 2015 [adults][10]	Randomised controlled trial	Primary schools in urban Changzhi, northern China	May 2013-Dec 2013	School-EduSalt: fifth-graders (mean age 10.1 years, SD=0.5) and adult members of their families (mean age 43.8 years, SD=12.2)	Control group - adults (baseline)	273	215.1±6.7	36±1	9.5±0.2	1636±61	Participant asked to do another 24h collection if missed one or more urine voids or spilt >10% of the total 24h urine volume
					Intervention group - adults (baseline)	275	167.13±11.46	45.41±5.09	9.85±0.72	1200±27	
Han et al, 2015[47]	Cross-sectional	Department of Hypertension at Peking University People's Hospital, Beijing	Mar 2010-Feb 2012	Regular hypertensive visitors, mean age 58.4 years (SD=14.5)		222	147.9±4.15		11.0±0.04		Complete 24-hour urine collection was defined as urine volume ≥500 ml as measured by a technician, recorded collection of ≥20 hours, and reports of spilling urine or missing a

											void no more than once in 24 hours
Wang et al, 2016[48]	Randomised controlled trial	Northern rural China: Hebei, Liaoning, Shanxi, Shaanxi, Ningxia Autonomous Region	May 2011-Nov 2012	CRHI-SRS controls: mean age 53.9 years (SD=14.1)	Controls	928	250.5±3.08	45.4±0.62			Urine collections excluded if participants reported missing more than one void, a collection period less than 22 h or longer than 26 h, suspected spillage of more than 10 % of the total volume, volume < 500 ml or > 6000 ml, urinary creatinine < 4.0 mmol/day or > 25 mmol/day for women or urinary creatinine < 6.0 mmol/day or > 30 mmol/day for men
Zhang et al, 2016[49]	Crossover trial	Northern China	NR	Rural community, mean age 50.6 years (SD=2.1)	Baseline	38	175.8±1.80				NR
Yongqing et al, 2016[50]	Cross-sectional	Jiangsu Province	Dec 2013-May 2014	Mean age 41.55 years (SD=13.797)	Male	1069	196.36±2.21	28.37±0.31			Assessed based on creatinine excretion
					Female	1133	180.47±1.95	27.64±0.27			
					Urban	823	205.23±2.49	29.97±0.35			
					Rural	1379	178.02±1.78	26.81±0.24			
					Aged 18-34 years	625	189.55±2.8	26.47±0.35			
					Aged 35-49 years	731	190.91±2.65	28.34±0.37			
					Aged 50-59 years	846	184.82±2.31	28.81±0.33			

					Total	2202	188.19±1.48	27.99±0.2			
Peng et al, 2016[51]	Cross-sectional	Shanxi Province	NR	PURE substudy: mean age 53.16 years, SD=8.09		116	157.93±4.65	27.59±1.09		1869±76	Participants with incomplete urine collections or missing data were excluded from this analysis
Zheng et al, 2017[52]	Crossover trial	Northern China	NR	Rural community, mean age 52.2 years (SD=1.8) in salt-sensitive subjects, 50.8 years (SD=2.4) in salt-resistant subjects	Salt-sensitive subjects (baseline)	13	156.2±13.9	29±2.4			NR
					Salt-resistant subjects (baseline)	25	175.3±12.9	32±1.6			
Guo et al, 2017[53]	Crossover trial	Rural area of Shaanxi Province	Jul-Aug 2013	Mean age 51.3 years (SD=2.5) in salt-sensitive subjects, 49.6 years (SD=1.4) in non-salt-sensitive subjects	Salt-sensitive subjects (baseline)	14	167.13±11.46	45.41±5.09	9.85±0.72	1200±27	NR
					Non-salt-sensitive subjects (baseline)	35	177.07±12.53	48.04±3.56	9.51±0.49	1160±12	
					Baseline	38	180.53±8.47	40.91±0.85	9.93±0.18	1445±32	
Li et al, 2017[54]	Cross-sectional	Shenyang, Jinan, Chengdu, Chongqing	NR	Mean age 39.0 years (SD=10.5) in low-salt preference group, 39.7 years (SD=9.8) medium-salt preference group, 44.0 years (SD=8.4) in high-salt preference group	Low-Salt Preference Group	416	191.4±4.03				NR
					Medium-Salt Preference Group	94	221.9±9.33				
					High-Salt Preference Group	96	243.2±8.18				
Deng et al, 2017[55]	Cross-sectional	Shanghai, Chongqing, Harbin, Shaoyang,	May 2013-Jul 2014	Han adults: mean age 48.86 years (SD=16.25) in the standard weight	Standard weight adults	376	207.93±52.97	41.41±2.82	9.46±0.55	1296±87	Exclusion: incomplete urine samples (urine
					Under-weight adults	24	194.79±6.75	47.54±1.89	9.95±0.23	1523±55	

		Lanzhou, Changshi		group, 53.96 years (SD=19.28) in the underweight group, 52.14 years (SD=14.25) in the overweight group, 50.03 years (SD=12.84) in the obese group	Overweight adults	149	192.66±11.96	45.66±3.32	10.46±0.45	1451±114	creatinine < 600 μg/24 h)
					Obese adults	35	193.4±6.7	36.1±1	9.3±0.2	1577±61	
Ma et al, 2017[56]	Cross-sectional	Rural areas of Chenggu and Qishan counties, Shaanxi Province	Feb 2015-Feb 2016	SSaSS substudy: with elevated risk of stroke, mean age 67.5 years (SD=6.8)		365	122.4±2.51	24.7±0.65	6.43±0.14	1419±29	Excluded from analysis if collection time fell outside the range of 22–26 h, total 24h urine volume was less than 500 mL or greater than 6000 mL, and 24h creatinine excretion was less than 3 mmol or greater than 25 mmol in women or less than 6 mmol or greater than 30 mmol in men
Zhou et al, 2017[57]	Cross-sectional	Dexing City, Jiangsi Province	NR	Mean age 51.1 years (SD=8.2)		141	155.4±3.6	28.1±0.83	5.6±0.19	1487±56	Excluded if an incomplete 24h urine collection was reported, the collection time fell outside the range of 22–26 h, or the total volume of urine was <500 mL
Dong et al, 2017[58]	Cross-sectional	Chenghai district, Longhu district and	Mar-Nov 2016	Mean age 56.3 years (SD=17.4)	Male	128.12±8.77	43.21±4.22	8.53±0.36	1792±115		Excluded: urine volume less than 500ml/24h, missed 1 void, 24h urine

		Jinping distict in Shantou city			Female	131.77±4.94	48.09±2.08	7.16±0.21	1663±51		creatinine <4 mmol (in women) or <6 mmol (in men)
Wang et al, 2018[59]	Crossover trial	Liquan and Lantian Counties, Shaanxi Province	NR	Mean age 50.5 years (SD=1.1)	Baseline	90	172.1±7.6	37.9±2			Any urine collection less than 500 mL or with a creatinine excretion lower than the population mean minus two standard deviations was discarded
Hu et al, 2018[60]	Crossover trial	Lantian, Shaanxi Province	NR	Rural community, mean age 51.2 years (SD=12.4)	Baseline	44	168.98±11.61	27.79±1.61			NR
Duan et al, 2018[61]	Cross-sectional	Cities of Tianjin and Luoyang	NR	Healthy lactating women aged 20-39 years	30	160.08±11.85				NR	Duan et al, 2018[61]

NR: not reported; SD: standard deviation; SE: standard error.

CRHI-SRS: China Rural Health Initiative Sodium Reduction Study; GenSalt: Genetic Epidemiology Network of Salt Sensitivity; INTERMAP: International Study of Macro-and Micro-Nutrients; PURE: Prospective Urban and Rural Epidemiological; School Edu-Salt: School-based Education Program to Reduce Salt Intake in Children and Their Families; SMASH: Shandong and Ministry of Health Action on Salt and Hypertension; SSaSS: Salt Substitute and Stroke Study; WHO CARDIAC: World Health Organization Cardiovascular Diseases and Alimentary Comparison.

Table S2. Mean sodium excretion (mmol/24h) for subgroups of studies – sensitivity analyses

	Base analysis	Excluding hospital-based studies	Excluding hypertensive participants	Excluding ethnic minorities	Placing Tibet in southern China
**Age groups**					
- 3–6 years	86.99 (69.88–104.1)	86.99 (69.88–104.1)	86.99 (69.88–104.1)	86.99 (69.88–104.1)	–
- 6–16 years	151.09 (131.55–170.63)	144.46 (123.53–165.39)	146.99 (128.51–165.46)	145.58 (126.41–164.76)	–
- >16 years	189.07 (182.14–195.99)	187.28 (180.24–194.33)	187.01 (180.26–193.75)	190.58 (183.61–197.56)	–
**Sex**					
- Female	181.54 (167.10– 195.99)	178.39 (164.50– 192.29)	166.08 (152.65– 179.51)	181.54 (167.10– 195.99)	–
- Male	194.76 (179.27– 210.25)	187.91 (172.11– 203.70)	181.57 (166.97– 196.17)	202.57 (187.40– 217.74)	–
**Geographical location**					
- Northern China	205.81 (193.15– 218.46)	202.33 (189.61– 215.06)	208.05 (195.18– 220.92)	205.43 (192.75– 218.11)	205.13 (192.21– 218.04)
- Southern China	156.97 (145.96– 167.99)	157.54 (144.90– 170.18)	157.92 (146.73– 169.10)	158.84 (147.94– 169.75)	161.45 (150.52– 172.39)
**Rigour of 24h urine collection**					
- Completeness not assessed / reported	188.43 (172.96– 203.90)	186.15 (170.41– 201.89)	181.95 (168.70– 195.20)	187.88 (172.34– 203.43)	–
- Completeness assessed	188.04 (175.56– 200.52)	185.50 (172.41– 198.59)	193.42 (180.78– 206.06)	189.09 (176.71– 201.48)	–

Table S2 (continued)

	Base analysis	Excluding hospital-based studies	Excluding hypertensive participants	Excluding ethnic minorities	Placing Tibet in southern China
**Study year (whole of China)**					
- 1980s	192.84 (159.15– 226.54)	179.06 (152.46– 205.66)	192.84 (159.15– 226.54)	194.76 (166.82– 222.70)	–
- 1990s	191.20 (167.79– 214.62)	188.16 (165.10– 211.21)	190.48 (165.47– 215.49)	191.65 (167.80– 215.50)	–
- 2000s	201.07 (164.10– 238.04)	201.07 (164.10– 238.04)	178.23 (125.28– 231.18)	201.07 (164.10– 238.04)	–
- 2010s	181.51 (169.93– 193.09)	181.51 (169.93– 193.09)	186.80 (176.90– 196.70)	181.51 (169.93– 193.09)	–
**Study year (northern China)**					
- 1980s	222.49 (210.68– 234.30)	217.69 (205.53– 229.85)	222.49 (210.68– 234.30)	218.60 (205.45– 231.74)	222.49 (210.68– 234.30)
- 1990s	228.97 (211.68– 246.25)	223.90 (203.02– 244.77)	230.94 (213.46– 248.42)	232.46 (215.29– 249.63)	225.89 (207.43– 244.35)
- 2000s	242.95 (238.85– 247.06)	242.95 (238.85– 247.06)	238.36 (233.17– 243.55)	242.95 (238.85– 247.06)	242.95 (238.85– 247.06)
- 2010s	194.53 (187.35– 201.71)	194.53 (187.35– 201.71)	197.56 (190.39– 204.72)	194.53 (187.35– 201.71)	194.53 (187.35– 201.71)
**Study year (southern China)**					
- 1980s	152.51 (138.05– 166.97)	152.55 (134.59– 170.51)	152.51 (138.05– 166.97)	168.16 (158.35– 177.98)	152.51 (138.05– 166.97)
- 1990s	150.36 (140.88– 159.83)	150.82 (141.21– 160.42)	149.34 (139.99– 158.70)	150.36 (140.88– 159.83)	155.81 (145.13– 166.48)
- 2000s	195.99 (169.50– 222.49)	195.99 (169.50– 222.49)	167.00 (153.29– 180.70)	195.99 (169.50– 222.49)	195.99 (169.50– 222.49)
- 2010s	178.15 (169.47– 186.82)	178.15 (169.47– 186.82)	180.13 (173.34– 186.92)	178.15 (169.47– 186.82)	178.15 (169.47– 186.82)

Table S3. Mean potassium excretion (mmol/24h) for subgroups of studies – sensitivity analyses

	Base analysis	Excluding hospital-based studies	Excluding hypertensive participants	Excluding ethnic minorities	Placing Tibet in southern China
**Age groups**					
- 3–6 years	14.65 (11.1–18.2)	14.65 (11.1–18.2)	14.65 (11.1–18.2)	14.65 (11.1–18.2)	–
- 6–16 years	25.23 (22.37–28.1)	26.03 (22.08–29.99)	26.03 (22.08–29.99)	24.35 (21.52–27.18)	–
- >16 years	36.35 (35.11 to 37.59)	35.97 (34.73 to 37.22)	36.02 (34.83 to 37.22)	36.09 (34.82 to 37.36)	–
**Sex**					
- Female	36.76 (33.37–40.15)	36.01 (32.69–39.33)	35.33 (32.51–38.16)	36.76 (33.37–40.15)	–
- Male	38.26 (35.65–40.86)	37.95 (35.35–40.56)	38.65 (36.05–41.26)	36.65 (33.88–39.41)	–
**Geographical location**					
- Northern China	38.19 (35.16–41.21)	38.00 (34.90–41.10)	38.42 (34.89–41.95)	38.62 (35.60–41.63)	38.14 (35.06–41.21)
- Southern China	36.66 (33.01–40.32)	36.67 (32.53–40.81)	35.74 (32.78–38.70)	36.13 (32.56–39.71)	36.80 (33.23–40.37)
**Rigour of 24h urine collection**					
- Completeness not assessed / reported	37.13 (33.53–40.73)	36.94 (33.24–40.63)	37.53 (33.02–42.03)	37.83 (34.24–41.41)	–
- Completeness assessed	37.45 (34.55–40.34)	37.38 (34.22–40.53)	36.65 (34.01–39.29)	37.11 (34.25–39.97)	–

Table S3 (continued)

	Base analysis	Excluding hospital-based studies	Excluding hypertensive participants	Excluding ethnic minorities	Placing Tibet in southern China
**Study year (whole of China)**					
- 1980s	36.98 (33.86– 40.11)	40.45 (33.02– 47.88)	36.98 (33.86– 40.11)	38.26 (33.82– 42.70)	–
- 1990s	37.34 (33.40– 41.27)	36.78 (32.83– 40.74)	37.83 (33.62– 42.04)	37.54 (33.71– 41.38)	–
- 2000s	33.52 (26.95– 40.08)	33.52 (26.95– 40.08)	31.05 (23.69– 38.41)	33.52 (26.95– 40.08)	–
- 2010s	37.96 (34.04–41.88)	37.96 (34.04–41.88)	36.82 (33.33– 40.32)	37.96 (34.04–41.88)	–
**Study year (northern China)**					
- 1980s	39.27 (37.16– 41.38)	39.49 (37.22– 41.76)	39.27 (37.16– 41.38)	39.42 (37.01– 41.82)	39.27 (37.16– 41.38)
- 1990s	38.53 (34.80– 42.26)	37.14 (33.09– 41.20)	38.72 (34.74– 42.70)	39.93 (37.39– 42.47)	38.45 (34.58– 42.32)
- 2000s	36.17 (33.74– 38.61)	36.17 (33.74– 38.61)	23.24 (22.30– 24.18)	36.17 (33.74– 38.61)	36.17 (33.74– 38.61)
- 2010s	38.82 (37.26–40.38)	38.82 (37.26–40.38)	38.97 (37.39– 40.54)	38.82 (37.26–40.38)	38.82 (37.26–40.38)
**Study year (southern China)**					
- 1980s	34.33 (31.86– 36.81)	33.94 (31.57– 36.30)	34.33 (31.86– 36.81)	30.46 (28.36– 32.57)	34.33 (31.86– 36.81)
- 1990s	35.41 (31.05– 39.76)	34.83 (30.62– 39.05)	36.75 (32.24– 41.27)	35.41 (31.05– 39.76)	35.65 (31.42– 39.88)
- 2000s	33.00 (27.82– 38.17)	33.00 (27.82– 38.17)	30.14 (24.33– 35.94)	33.00 (27.82– 38.17)	33.00 (27.82– 38.17)
- 2010s	35.23 (33.03– 37.42)	35.23 (33.03– 37.42)	33.14 (31.70– 34.58)	35.23 (33.03– 37.42)	35.23 (33.03– 37.42)

Table S4 Potential effect modifiers of adults' sodium excretion (mmol/24h) – sensitivity analyses

	Univariate		Multivariate	
	Slope (95% CI)	p-value	Slope (95% CI)	p-value
**Excluding hospital-based studies**				
Age (year)	0.25 (-0.59 to 1.1)	0.5553	0.11 (-0.77 to 0.98)	0.8121
Sex (% men)	0.45 (-0.09 to 0.98)	0.0994	0.25 (-0.28 to 0.78)	0.3452
Geographical location (each administrative region coded from South to North)	3.1 (2.05 to 4.16)	<0.0001	2.98 (1.77 to 4.20)	<0.0001
Rigour of 24h urine collection (not rigorous or not reported as reference)	-13.05 (-31.03 to 4.93)	0.1526	-0.15 (-0.99 to 0.69)	0.7219
Year of data collection (whole of China)	0.35 (-0.51 to 1.21)	0.4149	-9.13 (-26.55 to 8.28)	0.2999
Year of data collection (northern China only)	-1.21 (-2.26 to -0.17)	0.0239	–	–
Year of data collection (southern China only)	1.07 (-0.02 to 2.16)	0.0533	–	–
**Excluding hypertensive participants**				
Age (year)	1.03 (0.19 to 1.88)	0.0172	0.43 (-0.34 to 1.20)	0.2694
Sex (% men)	0.36 (-0.14 to 0.86)	0.1563	0.11 (-0.34 to 0.56)	0.6408
Geographical location (each administrative region coded from South to North)	3.31 (2.35 to 4.28)	<0.0001	3.38 (2.32 to 4.45)	<0.0001
Rigour of 24h urine collection (not rigorous or not reported as reference)	12.22 (-7.49 to 31.92)	0.2206	-0.54 (-1.24 to 0.17)	0.1331
Year of data collection (whole of China)	0.13 (-0.68 to 0.95)	0.7457	12.31 (-3.99 to 28.61)	0.1363
Year of data collection (northern China only)	-1.22 (-2.15 to -0.29)	0.0112	–	–
Year of data collection (southern China only)	0.76 (-0.04 to 1.56)	0.0603	–	–
**Excluding ethnic minorities**				
Age (year)	0.23 (-0.61 to 1.07)	0.5887	0.04 (-0.81 to 0.89)	0.9229
Sex (% men)	0.52 (0.05 to 0.99)	0.0312	0.25 (-0.23 to 0.74)	0.2996
Geographical location (each administrative region coded from South to North)	3.2 (2.19 to 4.21)	<0.0001	3.11 (1.94 to 4.28)	<0.0001
Rigour of 24h urine collection (not rigorous or not reported as reference)	-11.39 (-28.97 to 6.2)	0.2016	-0.22 (-1.01 to 0.58)	0.5877
Year of data collection (whole of China)	0.16 (-0.65 to 0.97)	0.6970	-6.59 (-23.26 to 10.08)	0.4345
Year of data collection (northern China only)	-1.21 (-2.14 to -0.28)	0.0117	–	–
Year of data collection (southern China only)	0.95 (-0.09 to 2.00)	0.0719	–	–
**Placing Tibet in southern China**				
Age (year)	0.25 (-0.59 to 1.09)	0.5573	0.08 (-0.77 to 0.94)	0.8491
Sex (% men)	0.53 (0.04 to 1.01)	0.0337	0.27 (-0.23 to 0.76)	0.2853
Geographical location (each administrative region coded from South to North)	3.25 (2.24 to 4.27)	<0.0001	3.15 (1.98 to 4.32)	<0.0001
Rigour of 24h urine collection (not rigorous or not reported as reference)	-12.47 (-30.22 to 5.29)	0.1665	-0.24 (-1.04 to 0.56)	0.5493
Year of data collection (whole of China)	0.18 (-0.64 to 0.99)	0.6723	-7.85 (-24.63 to 8.92)	0.3547
Year of data collection (northern China only)	-1.26 (-2.2 to -0.31)	0.0101	–	–
Year of data collection (southern China only)	0.93 (-0.2 to 2.06)	0.1033	–	–

Table S5 Potential effect modifiers of adults' potassium excretion (mmol/24h) – sensitivity analyses

	Univariate		Multivariate	
	Slope (95% CI)	p-value	Slope (95% CI)	p-value
**Excluding hospital-based studies**				
Age (year)	0.02 (-0.17 to 0.2)	0.8474	-0.07 (-0.31 to 0.17)	0.5445
Sex (% men)	0.01 (-0.12 to 0.14)	0.9029	0.00 (-0.16 to 0.16)	0.9982
Geographical location (each administrative region coded from South to North)	0.16 (-0.11 to 0.42)	0.2476	0.14 (-0.17 to 0.46)	0.3693
Rigour of 24h urine collection (not rigorous or not reported as reference)	1.24 (-2.74 to 5.21)	0.5379	1.77 (-2.73 to 6.27)	0.4347
Year of data collection (whole of China)	0.12 (-0.06 to 0.31)	0.1927	0.11 (-0.1 to 0.32)	0.2961
Year of data collection (northern China only)	0.00 (-0.25 to 0.25)	0.9858	–	–
Year of data collection (southern China only)	0.21 (-0.11 to 0.52)	0.1867	–	–
**Excluding hypertensive participants**				
Age (year)	0.01 (-0.21 to 0.22)	0.9532	-0.04 (-0.31 to 0.23)	0.7616
Sex (% men)	0.08 (-0.06 to 0.22)	0.2842	0.08 (-0.09 to 0.24)	0.3680
Geographical location (each administrative region coded from South to North)	0.21 (-0.08 to 0.51)	0.1538	0.15 (-0.2 to 0.5)	0.3872
Rigour of 24h urine collection (not rigorous or not reported as reference)	1.02 (-4.11 to 6.16)	0.6914	1.25 (-4.38 to 6.88)	0.6585
Year of data collection (whole of China)	0.09 (-0.11 to 0.29)	0.3560	0.1 (-0.13 to 0.33)	0.3932
Year of data collection (northern China only)	0.01 (-0.27 to 0.28)	0.9702	–	–
Year of data collection (southern China only)	0.12 (-0.2 to 0.44)	0.4515	–	–
**Excluding ethnic minorities**				
Age (year)	0.04 (-0.14 to 0.22)	0.6547	-0.03 (-0.27 to 0.2)	0.7773
Sex (% men)	0 (-0.11 to 0.12)	0.9483	0 (-0.15 to 0.14)	0.9725
Geographical location (each administrative region coded from South to North)	0.19 (-0.07 to 0.45)	0.1520	0.18 (-0.13 to 0.49)	0.2594
Rigour of 24h urine collection (not rigorous or not reported as reference)	0.56 (-3.37 to 4.48)	0.7785	0.95 (-3.45 to 5.35)	0.6685
Year of data collection (whole of China)	0.1 (-0.07 to 0.28)	0.2510	0.08 (-0.13 to 0.28)	0.4434
Year of data collection (northern China only)	-0.05 (-0.29 to 0.18)	0.6512	–	–
Year of data collection (southern China only)	0.23 (-0.07 to 0.53)	0.1244	–	–
**Placing Tibet in southern China**				
Age (year)	0.02 (-0.16 to 0.2)	0.8311	-0.07 (-0.31 to 0.16)	0.5363
Sex (% men)	0.01 (-0.11 to 0.13)	0.9054	0 (-0.15 to 0.15)	0.9900
Geographical location (each administrative region coded from South to North)	0.15 (-0.1 to 0.41)	0.2339	0.15 (-0.16 to 0.46)	0.3348
Rigour of 24h urine collection (not rigorous or not reported as reference)	1.18 (-2.68 to 5.04)	0.5444	1.84 (-2.53 to 6.21)	0.4041
Year of data collection (whole of China)	0.11 (-0.07 to 0.29)	0.2191	0.1 (-0.1 to 0.3)	0.3252
Year of data collection (northern China only)	-0.01 (-0.25 to 0.23)	0.9449	–	–
Year of data collection (southern China only)	0.19 (-0.11 to 0.49)	0.2005	–	–

Figure S1. Risk of bias in the included studies

A. Studies reporting sodium data

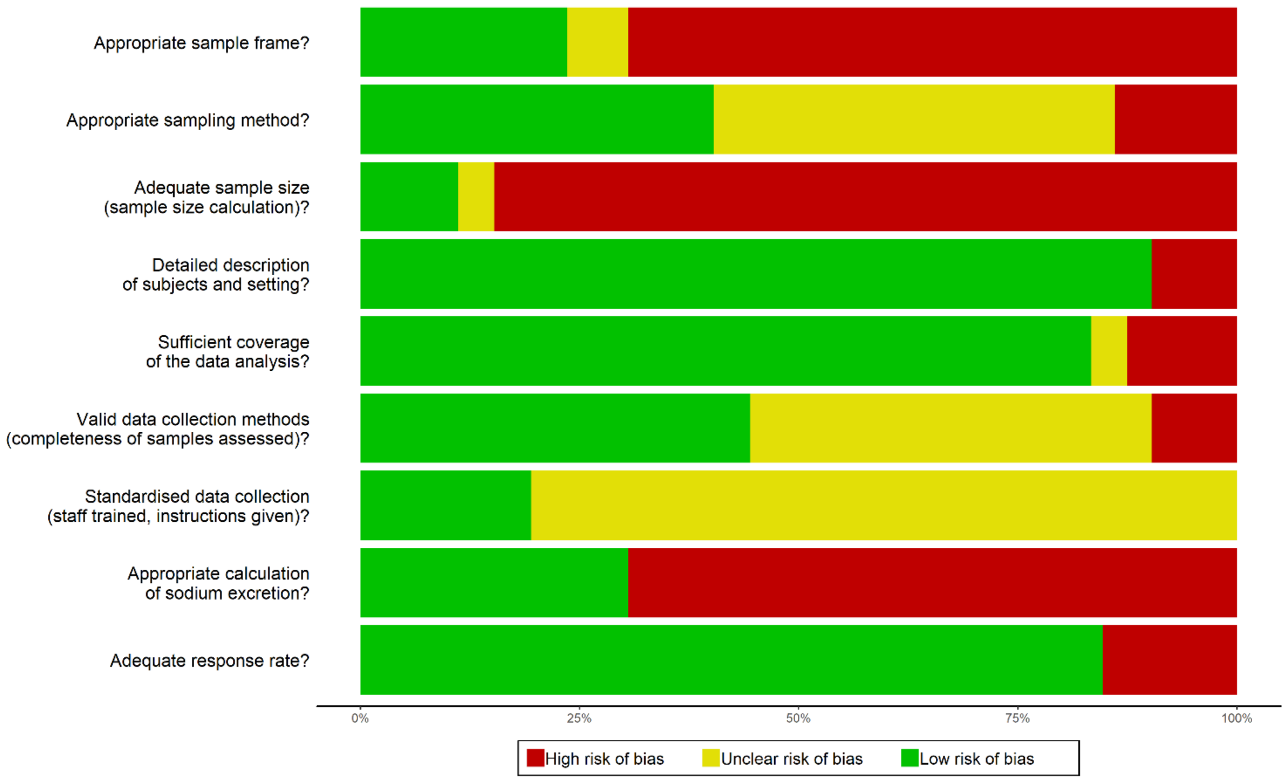

B. Studies reporting potassium data

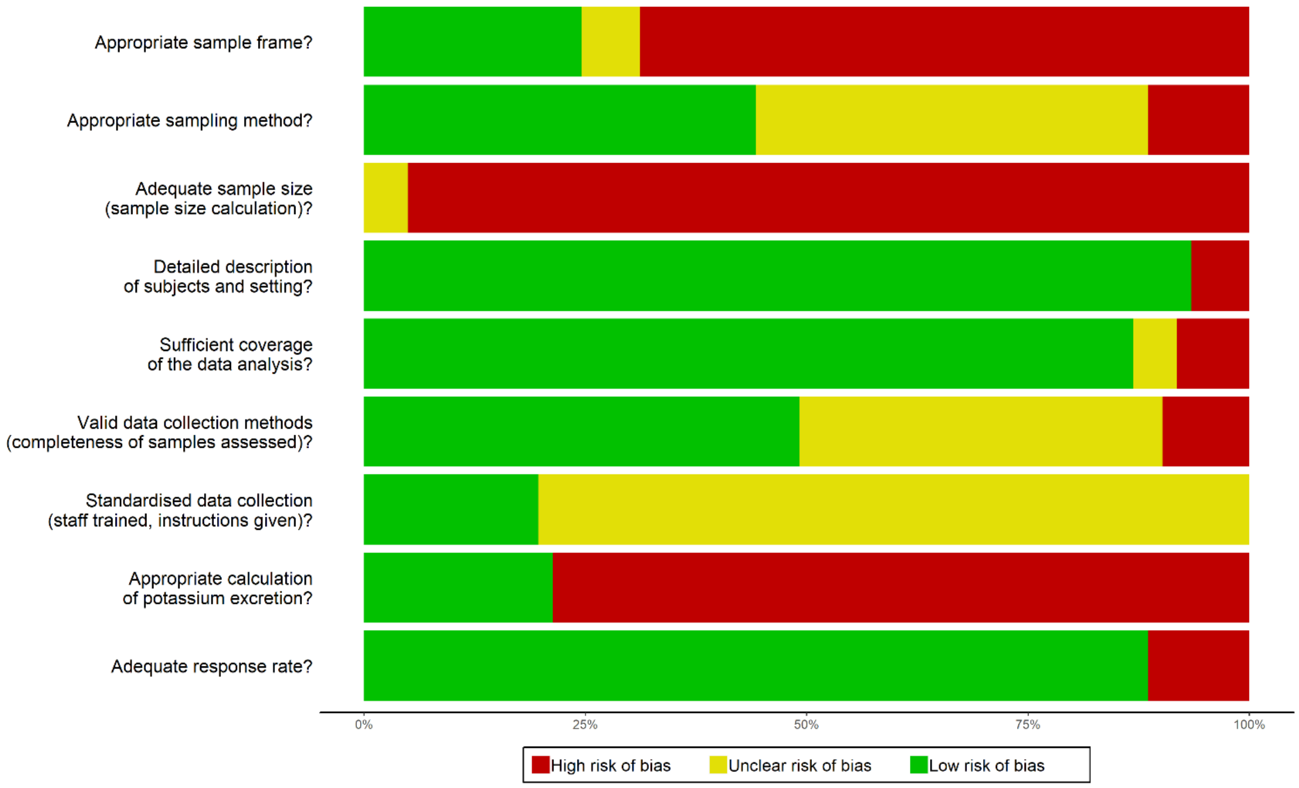

Grading details provided on the next page (text S2).

Supplemental References

1. Liu LS, Zhang KH, Wang J, Zhang XE, Wu HJ, Lin MQ, Gui RL, Du JH, Gu ML. Primary prevention of hypertension by sodium restriction. *Chinese Medical Journal*. 1987;100(11):899–902.
2. Yang Y. [Renal function of cations excretion in children predisposed to essential hypertension]. *Zhonghua Yu Fang Yi Xue Za Zhi [Chinese Journal of Preventive Medicine]*. 1991;25(3):152–154.
3. Zhu KM, He SP, Pan XQ, Zheng XR, Gu YA. The relation of urinary cations to blood pressure in boys aged seven to eight years. *American Journal of Epidemiology*. 1987;126(4):658–663.
4. Wu Y, Cai R, Zhou B, Xu X. Effects of genetic factors and dietary electrolytes on blood pressure of rural secondary school students in Hanzhong. *Chinese Medical Sciences Journal = Chung-Kuo I Hsueh K'o Hsueh Tsa Chih*. 1991;6(3):148–152.
5. Xu X.-J., Liang X.-H., Hu G.-M., Mao X.-M., Quanyangyi, Ozawa Y., Zhang X.-Y., Dilixiati, Maimaiti-Yasen. Ambulatory blood pressure and biochemical indicator analysis of 9-10 years old Kazakhstan Clan children in Xinjiang Baliken area. *Journal of Clinical Rehabilitative Tissue Engineering Research*. 2009;13(7):1379–1382.
6. Zhang Q, Liao Y, Tang C, Du J, Jin H. Twenty-four-hour urinary sodium excretion and postural orthostatic tachycardia syndrome. *The Journal of Pediatrics*. 2012;161(2):281–284.
7. Li J., Zhang Q., Liao Y., Zhang C., Du J. Clinical value of 24-hour urinary sodium determination in children with postural tachycardia syndrome. *Zhonghua er ke za zhi = Chinese journal of pediatrics*. 2015;53(3):203–207.
8. Zhang L., Zhao F., Zhang P., Gao J., Liu C., He F.J., Lin C.-P. A pilot study to validate a standardized one-week salt estimation method evaluating salt intake and its sources for family members in China. *Nutrients*. 2015;7(2):751–763.
9. Li J., Liao Y., Du J., Zhang Q. Relationship between 24-hour urinary sodium and renin-angiotensin-aldosterone system in children with postural tachycardia syndrome. *National Medical Journal of China*. 2015;95(36):2928–2932.
10. He FJ, Wu Y, Feng X-X, Ma J, Ma Y, Wang H, Zhang J, Yuan J, Lin C-P, Nowson C, MacGregor GA. School based education programme to reduce salt intake in children and their families (School-EduSalt): cluster randomised controlled trial. *BMJ (Clinical research ed.)*. 2015;350:h770.
11. Tsai TJ, Su CJ, Chen YM, Hsieh BS, Chen WY, Yen TS. Urinary kallikrein excretion in chronic renal disease with respect to salt intake and renal reserve. *Journal of the Formosan Medical Association = Taiwan Yi Zhi*. 1991;90(6):525–530.
12. Zhao GS, Yuan XY, Gong BQ, Wang SZ, Cheng ZH. Nutrition, metabolism, and hypertension. A comparative survey between dietary variables and blood pressure among three nationalities in China. *Journal of Clinical Hypertension*. 1986;2(2):124–131.
13. Liu LS, Zheng DY, Lai SH, Wang GQ, Zhang YL. Variability in 24-hour urine sodium excretion in Chinese adults. *Chinese Medical Journal*. 1986;99(5):424–426.
14. Kesteloot H, Huang DX, Li YL, Geboers J, Joossens JV. The relationship between cations and blood pressure in the People's Republic of China. *Hypertension (Dallas, Tex.: 1979)*. 1987;9(6):654–659.
15. Liu LS, Zheng DY, Jin L, Liao YL, Liu K, Stamler J. Variability of urinary sodium and potassium excretion in north Chinese men. *Journal of Hypertension*. 1987;5(3):331–335.
16. Intersalt Cooperative Research Group. Intersalt: an international study of electrolyte excretion and blood pressure. Results for 24 hour urinary sodium and potassium excretion. *BMJ : British Medical Journal*. 1988;297(6644):319–328.

17. He J, Tell GS, Tang YC, Mo PS, He GQ. Relation of electrolytes to blood pressure in men. The Yi people study. *Hypertension (Dallas, Tex.: 1979)*. 1991;17(3):378–385.
18. He J, Klag MJ, Whelton PK, Chen JY, Mo JP, Qian MC, Coresh J, Mo PS, He GQ. Agreement between overnight and 24-hour urinary cation excretions in southern Chinese men. *American Journal of Epidemiology*. 1993;137(11):1212–1220.
19. Chan EL, MacDonald D, Ho SC, Swaminathan R. Potassium intake and urinary calcium excretion in healthy subjects. *Mineral and Electrolyte Metabolism*. 1993;19(1):36–38.
20. Pan WH, Chen JY, Chen YC, Tsai WY. Diurnal electrolyte excretion pattern affects estimates of electrolyte status based on 24-hour, half-day, and overnight urine. *The Chinese Journal of Physiology*. 1994;37(1):49–53.
21. Tian HG, Nan Y, Shao RC, Dong QN, Hu G, Pietinen P, Nissinen A. Associations between blood pressure and dietary intake and urinary excretion of electrolytes in a Chinese population. *Journal of Hypertension*. 1995;13(1):49–56.
22. Hou R, Liu Z, Liu J. [The study of sympathetic nervous activity during the period of chronic salt loading in salt-sensitive subjects]. *Chinese Journal of Cardiology*. 1997;25(6):414–418.
23. Chan TY, Chan AY, Lau JT, Critchley JA. Sodium and potassium intakes and blood pressure in Chinese adults in Hong Kong: A comparison with southern China. *Asia Pacific Journal of Clinical Nutrition*. 1998;7(1):33–36.
24. Liu L, Mizushima S, Gao M. Body mass index, urinary sodium excretion, and blood pressure in seven Chinese populations: results from the WHO Cardiovascular Diseases and Alimentary Comparison Study. *CVD Prev*. 2000;3:11–17.
25. Cheung BM, Ho SP, Cheung AH, Lau CP. Diastolic blood pressure is related to urinary sodium excretion in hypertensive Chinese patients. *QJM: monthly journal of the Association of Physicians*. 2000;93(3):163–168.
26. Gu D, He J, Wu X, Duan X, Whelton PK. Effect of potassium supplementation on blood pressure in Chinese: a randomized, placebo-controlled trial. *Journal of Hypertension*. 2001;19(7):1325–1331.
27. Zhu D, Liu Z, Liu J, Liu Y. Renal endogenous ET-1 and urinary sodium excretion and microalbuminuria in human salt-sensitive hypertension. *Journal of Pharmaceutical Analysis*. 2001;13(1):30–32.
28. Jumabay M, Kawamura H, Mitsubayashi H, Ozawa Y, Izumi Y, Kasamaki Y, Shimabukuro H, Cheng Z, Aisa M, Wang S. Urinary electrolytes and hypertension in elderly Kazakhs. *Clinical and Experimental Nephrology*. 2001;5(4):217–221.
29. Xie J, Liu L, Kesteloot H. Blood pressure and urinary cations in a low-fat intake Chinese population sample. *Acta Cardiologica*. 2001;56(3):163–168.
30. Yamori Y, Liu L, Mu L, Zhao H, Pen Y, Hu Z, Kuga S, Negishi H, Ikeda K, Japan-China Cooperative Study Group: Chongqing Project. Diet-related factors, educational levels and blood pressure in a Chinese population sample: findings from the Japan-China Cooperative Research Project. *Hypertension Research: Official Journal of the Japanese Society of Hypertension*. 2002;25(4):559–564.
31. Zhao L, Stamler J, Yan LL, Zhou B, Wu Y, Liu K, Daviglus ML, Dennis BH, Elliott P, Ueshima H, Yang J, Zhu L, Guo D, INTERMAP Research Group. Blood pressure differences between northern and southern Chinese: role of dietary factors: the International Study on Macronutrients and Blood Pressure. *Hypertension (Dallas, Tex.: 1979)*. 2004;43(6):1332–1337.
32. Cheung DBMY, Law CY, McGhee SM, Ng PPY, Lau C-P, Kumana CR. The Relationship Between Sodium and Blood Pressure in Hong Kong Chinese. *Clinical Research and Regulatory Affairs*. 2004;21(2):145–154.

33. Zhou X., Liu J.-X., Shi R., Yang N., Song D.-L., Pang W., Li Y.-M. Compound ion salt, a novel low-sodium salt substitute: From animal study to community-based population trial. *American Journal of Hypertension*. 2009;22(9):934–942.
34. He J., Gu D., Chen J., Jaquish C.E., Rao D.C., Hixson J.E., Chen J.-C., Duan X., Huang J.-F., Chen C.-S., Kelly T.N., Bazzano L.A., Whelton P.K. Gender difference in blood pressure responses to dietary sodium intervention in the GenSalt study. *Journal of Hypertension*. 2009;27(1):48–54.
35. Zhang G, Ma J, Guo X, Dong J, Chen X, Zhang J, Su J, Tang J, Xu A. [Field observation on the effect of low-sodium and high-potassium salt substitute on blood pressure in the rural community-based population in China]. *Zhonghua Liu Xing Bing Xue Za Zhi*. 2011;32(9):859–863.
36. Liu Y, Wu J, Zhang L, Xu H, Liu Z, Lu J, Zhang J, Feng L, Guo Q, Zhao C, Liu J, Wei H, Cao S, Zhao H. [Influence factors of salt-sensitive hypertension and responses of blood pressure and urinary sodium and potassium excretion to acute oral saline loading among essential hypertensive patients]. *Zhonghua Xin Xue Guan Bing Za Zhi*. 2013;41(12):1015–1019.
37. Gu D, Zhao Q, Chen J, Chen J-C, Huang J, Bazzano LA, Lu F, Mu J, Li J, Cao J, Mills K, Chen C-S, Rice T, Hamm LL, He J. Reproducibility of blood pressure responses to dietary sodium and potassium interventions: the GenSalt study. *Hypertension (Dallas, Tex.: 1979)*. 2013;62(3):499–505.
38. Chen J, Tian Y, Liao Y, Yang S, Li Z, He C, Tu D, Sun X. Salt-Restriction-Spoon Improved the Salt Intake among Residents in China. *PLoS ONE*. 2013;8(11).
39. Chen J, Liao Y, Li Z, Tian Y, Yang S, Tu D, He C, Sun X. [Analysis of the determinants of salt-restriction behavior among urban and rural residents in Beijing with health belief model]. *Beijing Da Xue Xue Bao. Yi Xue Ban = Journal of Peking University. Health Sciences*. 2014;46(2):242–246.
40. Hu B.-C., Li Y., Liu M., Li L.-H., Sheng C.-S., Zhang Y., Wang J.-G. Blood pressure and urinary sodium excretion in relation to 16 genetic polymorphisms in the natriuretic peptide system in Chinese. *Endocrine Journal*. 2014;61(9):861–874.
41. Xu J., Wang M., Chen Y., Zhen B., Li J., Luan W., Ning F., Liu H., Ma J., Ma G. Estimation of salt intake by 24-hour urinary sodium excretion: a cross-sectional study in Yantai, China. *BMC public health*. 2014;14((Xu J.)):136.
42. Zhou X, Yuan F, Ji W-J, Guo Z-Z, Zhang L, Lu R-Y, Liu X, Liu H-M, Zhang W-C, Jiang T-M, Zhang Z, Li Y-M. High-salt intake induced visceral adipose tissue hypoxia and its association with circulating monocyte subsets in humans. *Obesity (Silver Spring, Md.)*. 2014;22(6):1470–1476.
43. Liu Z-M, Ho SC, Tang N, Chan R, Chen Y-M, Woo J. Urinary sodium excretion and dietary sources of sodium intake in Chinese postmenopausal women with prehypertension. *PloS One*. 2014;9(8):e104018.
44. Ge Z., Guo X., Chen X., Tang J., Yan L., Ren J., Zhang J., Lu Z., Dong J., Xu J., Cai X., Liang H., Ma J. Association between 24 h urinary sodium and potassium excretion and the metabolic syndrome in Chinese adults: The Shandong and Ministry of Health Action on Salt and Hypertension (SMASH) study. *British Journal of Nutrition*. 2015;113(6):996–1002.
45. Ge Z, Zhang J, Chen X, Guo X, Yan L, Tang J, Cai X, Xu J, Hou L, Ma J. [Association between 24 h urinary sodium to potassium ratio and metabolic syndrome in Chinese adults]. *Chinese Journal of Epidemiology*. 2015;36(8):790–793.
46. Wang Y, Mu JJ, Geng LK, Wang D, Ren KY, Guo TS, Chu C, Xie BQ, Liu FQ, Yuan ZY. Effect of salt intake and potassium supplementation on brachial-ankle pulse wave velocity in Chinese subjects: an interventional study. *Brazilian Journal of Medical and*

Biological Research = Revista Brasileira De Pesquisas Medicas E Biologicas. 2015;48(1):83–90.
47. Han W., Sun N., Chen Y., Wang H., Xi Y., Ma Z. Validation of the spot urine in evaluating 24-hour sodium excretion in Chinese hypertension patients. *American Journal of Hypertension*. 2015;28(11):1368–1375.
48. Wang X., Li X., Vaartjes I., Neal B., Bots M.L., Hoes A.W., Wu Y. Does education level affect the efficacy of a community based salt reduction program? - A post-hoc analysis of the China Rural Health Initiative Sodium Reduction Study (CRHI-SRS). *BMC public health*. 2016;16(1):759.
49. Zhang Y., Li F.X., Liu F.-Q., Chu C., Wang Y., Wang D., Guo T.-S., Wang J.-K., Guan G.-C., Ren K.-Y., Mu J.-J. Elevation of fasting ghrelin in healthy human subjects consuming a high-salt diet: A novel mechanism of obesity? *Nutrients*. 2016;8(6).
50. Yongqing Z., Ming W., Jian S., Pengfei L., Xiaoqun P., Meihua D., Peian L., Jianmei D., Guoyu Z., Jie Y., Ping L., Yan X. Prevalence, awareness, treatment and control of hypertension and sodium intake in Jiangsu Province, China: a baseline study in 2014. *BMC public health*. 2016;16((Yongqing Z.) Jiangsu provincial Center for Disease Control and Prevention, 210009, Nanjing, China. zyq6943@163.com):56.
51. Peng Y, Li W, Wang Y, Chen H, Bo J, Wang X, Liu L. Validation and Assessment of Three Methods to Estimate 24-h Urinary Sodium Excretion from Spot Urine Samples in Chinese Adults. *PloS One*. 2016;11(2):e0149655.
52. Zheng W-L, Chu C, Lv Y-B, Wang Y, Hu J-W, Ma Q, Yan Y, Cao Y-M, Dang X-L, Wang K-K, Mu J-J. Effect of Salt Intake on Serum Glucagon-Like Peptide-1 Levels in Normotensive Salt-Sensitive Subjects. *Kidney & Blood Pressure Research*. 2017;42(4):728–737.
53. Guo T-S, Dai Y, Ren K-Y, Mu J-J, Ren J, Wang D, Wang Y, Chu C, Li Y, Yuan Z-Y. Effects of salt loading and potassium supplement on the circadian blood pressure profile in salt-sensitive Chinese patients. *Blood Pressure Monitoring*. 2017;22(6):307–313.
54. Li Q, Cui Y, Jin R, Lang H, Yu H, Sun F, He C, Ma T, Li Y, Zhou X, Liu D, Jia H, Chen X, Zhu Z. Enjoyment of Spicy Flavor Enhances Central Salty-Taste Perception and Reduces Salt Intake and Blood Pressure. *Hypertension (Dallas, Tex.: 1979)*. 2017;70(6):1291–1299.
55. Deng T, Mai Z, Cai C, Duan X, Zhu W, Zhang T, Wu W, Zeng G. Influence of weight status on 24-hour urine composition in adults without urolithiasis: A nationwide study based on a Chinese Han population. *PloS One*. 2017;12(9):e0184655.
56. Ma W, Yin X, Zhang R, Liu F, Yang D, Fan Y, Rong J, Tian M, Yu Y. Validation and Assessment of Three Methods to Estimate 24-h Urinary Sodium Excretion from Spot Urine Samples in High-Risk Elder Patients of Stroke from the Rural Areas of Shaanxi Province. *International Journal of Environmental Research and Public Health*. 2017;14(10).
57. Zhou L, Tian Y, Fu J-J, Jiang Y-Y, Bai Y-M, Zhang Z-H, Hu X-H, Lian H-W, Guo M, Yang Z-X, Zhao L-C. Validation of spot urine in predicting 24-h sodium excretion at the individual level. *The American Journal of Clinical Nutrition*. 2017;105(6):1291–1296.
58. Dong W, Zhang Q, Jiang J, Chen H, Chen X, Shao S, Liu J, Ji Y. [Evaluating the sodium intake of community residents in Shantou city by 24-h urine method]. *Chin J Prev Contr Chron Di*. 2017;25(7):481–484.
59. Wang Y, Chu C, Wang K-K, Hu J-W, Yan Y, Lv Y-B, Cao Y-M, Zheng W-L, Dang X-L, Xu J-T, Chen W, Yuan Z-Y, Mu J-J. Effect of Salt Intake on Plasma and Urinary Uric Acid Levels in Chinese Adults: An Interventional Trial. *Scientific Reports*. 2018;8(1):1434.

60. Hu J-W, Wang Y, Chu C, Mu J-J. Effect of Salt Intervention on Serum Levels of Fibroblast Growth Factor 23 (FGF23) in Chinese Adults: An Intervention Study. *Medical Science Monitor: International Medical Journal of Experimental and Clinical Research*. 2018;24:1948–1954.
61. Duan L, Liu W, Zhang P, Liu S, Liu X, Sang M, Liu L, Lin H, Sang Z. Salt Intake of Lactating Women as Assessed by Modified Food Weighted Records. *Journal of the American College of Nutrition*. 2018;37(7):614–619.
